# Marine Bioactive Molecules as Inhibitors of the Janus Kinases: A Comparative Molecular Docking and Molecular Dynamics Simulation Approach

**DOI:** 10.3390/cimb46090631

**Published:** 2024-09-23

**Authors:** Emad A. Ahmed, Salah A. Abdelsalam

**Affiliations:** 1 Department of Biological Sciences, College of Science, King Faisal University, Hofouf 31982, Saudi Arabia; 2 Lab of Molecular Physiology, Zoology Department, Faculty of Science, Assiut University, Assiut 71516, Egypt; sabdelraouf@kfu.edu.sa; 3 Zoology Department, Faculty of Science, Assiut University, Assiut 71516, Egypt

**Keywords:** marine biomolecules, rheumatoid arthritis, Janus kinases, molecular docking, molecular dynamics simulation

## Abstract

A treasure trove of naturally occurring biomolecules can be obtained from sea living organisms to be used as potential antioxidant and anti-inflammatory agents. These bioactive molecules can target signaling molecules involved in the severity of chronic autoimmune diseases such as rheumatoid arthritis (RA). The intracellular tyrosine kinases family, Janus kinases (JAKs, includes JAK1, JAK2, and JAK3), is implicated in the pathogenesis of RA through regulating several cytokines and inflammatory processes. In the present study, we conducted molecular docking and structural analysis investigations to explore the role of a set of bioactive molecules from marine sources that can be used as JAKs’ specific inhibitors. Around 200 antioxidants and anti-inflammatory molecules out of thousands of marine molecules found at the Comprehensive Marine Natural Products Database (CMNPD) website, were used in that analysis. The details of the interacting residues were compared to the recent FDA approved inhibitors tofacitinib and baricitinib for data validation. The shortlisted critical amino acids residues of our pharmacophore-based virtual screening were LYS905, GLU957, LEU959, and ASP1003 at JAK1, GLU930 and LEU932 at JAK2, and GLU905 and CYS909 of JAK3. Interestingly, marine biomolecules such as Sargachromanol G, Isopseudopterosin E, Seco-Pseudopterosin, and CID 10071610 showed specific binding and significantly higher binding energy to JAK1 active/potential sites when being compared with the approved inhibitors. In addition, Zoanthoxanthin and Fuscoside E bind to JAK2′s critical residues, GLU930 and LEU932. Moreover, Phorbaketal and Fuscoside E appear to be potential candidates that can inhibit JAK3 activity. These results were validated using molecular dynamics simulation for the docked complexes, JAK1(6sm8)/SG, JAK2 (3jy9)/ZAX, and JAK3 (6pjc)/Fuscoside E, where stable and lower binding energy were found based on analyzing set of parameters, discussed below (videos are attached). A promising role of these marine bioactive molecules can be confirmed in prospective preclinical/clinical investigations using rheumatoid arthritis models.

## 1. Introduction

The chronic autoimmune inflammatory condition rheumatoid arthritis (RA) occurs due to complex etiological reasons. At the pathophysiological level, RA is manifested by infiltrated immune cells, and synovial lining hyperplasia leading to destruction of articular cartilage and bone [1,2,3]. Fortunately, recent years’ studies have enabled us to understand the pathophysiology of RA and have provided more details about the treatment protocols and strategies [4,5,6]. In this regard, several preclinical and clinical trials have been recently conducted to test the chemotherapeutic effect of various agents in RA [5,6,7]. The treatment strategies of RA rely on using inhibitors that can interact with the active immune system molecules and, hence, reduce the chronic inflammatory state. These therapeutic inhibitors include anti-inflammatory and analgesic drugs, glucocorticoids, immunosuppressive agents, and disease-modifying antirheumatic drugs (DMARDs). Of these drugs, the inhibitors of the intracellular tyrosine kinases Janus kinases (JAKs) are the latest class of DMARDs to emerge in treating RA [8,9]. The family of nonreceptor protein kinases, the Janus kinases, is involved in regulating cytokines and growth factors signaling pathways through catalyzing tyrosine residues in ATP-dependent phosphorylation on substrates, and, hence, regulate immune cells’ function and oncogenesis. Janus kinases include four members, JAK1, JAK2, JAK3, and the tyrosine kinase 2. Structurally, each JAK protein forms seven distinct JAK homology (JH1-JH7) domains, from N to C terminus. Each kinase is composed of seven homology domains (JH), of which JH1 is the kinase domain and JH2 is the pseudokinase domain, while the SH2 domain is composed of part of JH3 and JH4 and JH5, the JH6, and part of the JH4 domain form the FERM domain [8,10,11].

Tofacitinib, the first JAK inhibitor to be studied and approved (by FDA) in humans for treating RA and other autoimmune diseases, can efficiently inhibit JAK1 and JAK3 and, to a lower extent, JAK2 [8,9]. Upon administrating orally, tofacitinib can bind to JAKs and thus prevents the activation of the JAK-signal transducers and activators of transcription (STAT) signaling pathway. On the other hand, the selective oral inhibitor of JAK1 and JAK2, baricitinib, was obtained through modifying the structure of tofacitinib. Baricitinib showed efficient therapeutic effect in several autoimmune diseases, including RA and other diseases, through worsening of symptoms and reducing the inflammatory state [12]. The conserved residues of glutamate and leucine amino acids, located in the hinge region of JAKs, were reported to stabilize tofacitinib and other inhibitors through the formation of strong hydrogen bonds. These include GLU957 and LEU959 of JAK1, GLU930 and LEU932 of JAK2, and LEU903 and GLU905 of JAK3 [13]. However, the CYS909 amino acid residue on the JAK3 protein was considered as a potential target for the covalent attachment of inhibitors [14].

Related to the above, recent attention has been given to the molecular treasure of natural biomolecules of antioxidant and anti-inflammatory agents that can be obtained from the seawater living organisms [15,16]. However, several potential marine anti-inflammatory agents can still be tested preclinically and clinically to be used in the treatment of arthritis.

In the present study, thousands of nontoxic marine molecules at the CMNPD library website, https://www.cmnpd.org accessed on 20 March 2023, were screened. Those compounds having antioxidant or anti-inflammatory properties and molecular weight lower than 480 g/mol were selected. After analyzing SwissADME software results (http://www.swissadme.ch , accessed on 20 March 2023), the binding affinities of these molecules to the Janus kinases JAK1, JAK2, and JAK3 were investigated virtually using molecular docking analysis. To relate the binding-based inhibitory action of these marine bioactive molecules to RA, the relative structural interaction was evaluated and compared to the recent FDA-approved inhibitors tofacitinib and baricitinib. Interestingly, molecules such as Fuscoside E, Sargachromanol G, Isopseudopterosin E, Seco-Pseudopterosin, and Zoanthoxanthin were found to have stable and higher binding affinities to JAKs than the approved inhibitors. The binding affinities and the specificity were confirmed using molecular dynamics simulation for 30 ns of some of these compounds. Future analysis of the preclinical and clinical effects of the highlighted molecules may clarify the inhibitory therapeutic role of these marine biomolecules in treating RA. 

## 2. Materials and Methods

### 2.1. Virtual Screening of Marine Antioxidant and Anti-Inflammatory Molecules

In the current study, more than 24.000 marine biomolecules listed at the Comprehensive Marine Natural Products Database website, https://www.cmnpd.org, Beijing, P.R. China/ (accessed 20 January 2023), were filtered for being nontoxic, highly reactive, and having molecular weight between 200 and 500 g/mol, where 732 were obtained. Other useful highly reactive marine biomolecules identified after 2020 (based on PubMed search) were also checked. Of these compounds, more than 200 nontoxic compounds having antioxidant or anti-inflammatory properties were selected to be tested and screened for water solubility, lipophilicity, optimal behavioral, physiochemical properties, pharmacokinetics, and drug-likeness characteristics. That was conducted using the SwissADME online tool, http://www.swissadme.ch, accessed on 20 March 2023, Lausanne, Switzerland, where 70 molecules were selected to be tested for in silico molecular docking. Lipinski’s rule was applied in which these orally active drugs have no more than one violation of the following characteristics: ten or fewer hydrogen bond acceptors, five or fewer hydrogen bond donors, a molecular mass less than 500 g/mol, and an estimated octanol-water partition coefficient (Clog P) lower than 5. In addition, the screening included a selective property based on binding to specific amino acids residues (active and /or ATP binding sites), listed and summarized in Table 1, and representative figures are shown as unpublished data.

### 2.2. Preparation of 3D Protein Structure

The 3D structure of JAKs was obtained from the Protein Data Bank (PDB, https://www.rcsb.org, USA). The retrieved kinases include 6sm8 (JAK1), 3jy9 (JAK2), and 6pjc (JAK3). The ligand–protein complexes were prepared and purified using discovery studio and the Swiss pdb viewer software. Details of the ligand purification, heteroatom deletion, and energy minimization are provided in Supplementary Materials. Briefly, at the PDB website, the protein structure, sequence length, chains, and bounded native ligands were analyzed. After downloading the pdb file from the PDB database, discovery studio was used to select and delete the water molecules, the hetero atoms, and the bound ligands. The text of the protein was purified from any coordinates before being treated using Swiss pdb viewer for energy minimization and purification, as indicated in the Supplementary Materials. A representative figure showing JAK proteins (JAK1, JAK2, and JAK3) domains indicating the active residues and the ATP binding sites was evaluated using CASTp 3.0 software. The protein’s topography, structures, and pockets, as well as functional sites, were obtained and analyzed using the freely accessible web server at http://sts.bioe.uic.edu/castp/, accessed on 1 April 2023.

### 2.3. Preparation of Ligand Structure

As shown above, 70 molecules were selected after computing the physicochemistry and estimating the pharmacokinetics and medicinal chemistry friendliness of the marine biomolecules. Then, chemical structures of these ligands were retrieved from the PubChem compound database (http://www.ncbi.nlm.nih.gov/search, Bethesda, MD, USA). The above steps of preparing and purifying the protein and the ligands were conducted between January and May 2023. The retrieved ligand structures in sdf format were used directly for protein-–ligand interaction at the cb dock2 website.

### 2.4. Binding Pocket and Molecular Docking

For drug discovery using computational analysis, protein and ligand 3D structures were used to predict binding sites and affinities [17,18]. The binding length, the established binding pockets, and the involved amino acid’ sequences at the ligand binding areas of the protein surface, in addition to the binding energy, were conducted using curvature-based cavity detection and molecular docking (Auto-Dock Vina-based procedure) in the CB-Dock server, https://cadd.labshare.cn/cb-dock2/php/index.php, Chengdu, China, accessed on 2023 starting from 10 January 2023.

### 2.5. Discovery Studio and Scoring the Binding Affinity, Distance and Sites

The molecular docking result of ligand protein interactions, hydrophobic interactions between H-H and H-C, as well as other types of interactions and binding sites, were investigated using the discovery studio software, Discovery studio 2021 client. The distance between H-H and H-C (the bond length) was measured in angstrom (Å). Other analyzed interaction residues are provided as unpublished materials. 

### 2.6. Molecular Dynamics Simulation

Molecular dynamics simulation of the docked ligand protein interactions was conducted according to Lemkul, [19]; the GROMACS tutorial can be accessed at http://www.mdtutorials.com/gmx/complex/index.html, Blacksburg, Virginia USA, accessed on 25 April 2023. The Ubuntu system for Windows was used to run the GROMACS tutorials. The installed software is listed in the Supplementary Materials. The used protocol and the tutorial procedures were modified according to the error’s correction and the duration of the MD running time (30 ns). Parameters and software were used to estimate the behavior of the complex at lower energy, such as discovery studio and chimera 1.17.3, and the root-mean-square deviation (RMSD) and the root-mean-square fluctuation (RMSF). These parameters were obtained using the Ubuntu- GROMACS codes. Other calculations, including kinetics, the potential and the total energies, as well as the H-bonds formation, were also estimated. Details about the outcomes of these estimated parameters are shown in the Supplementary Materials.

## 3. Results

### 3.1. Active and ATP-Binding Sites at JAK Proteins

**Figure 1:**
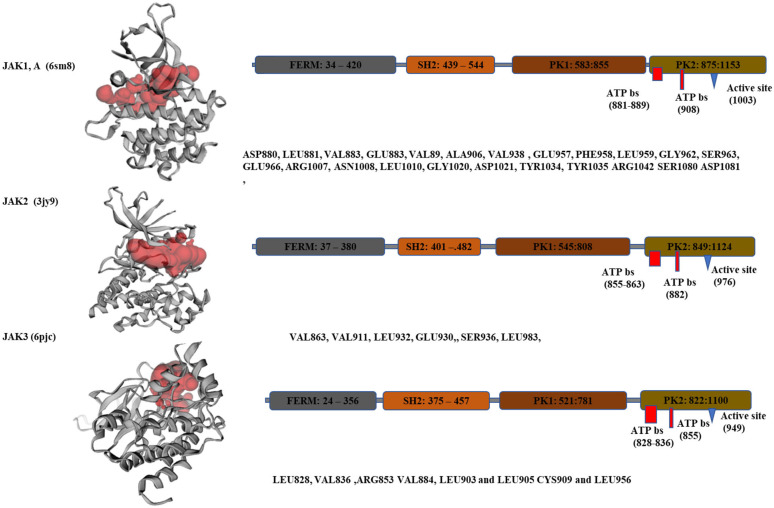
The structure, the active sites, and the conserved (ATP binding) phosphorylation sites of the JAK family proteins. Details of the JAK proteins topography, structures, pockets, and functional sites were retrieved from the CASTp 3.0 web server.

The structure, the active sites, and the conserved (ATP-binding) phosphorylation sites of the JAK family proteins are shown in Figure 1. Details of the JAK proteins binding pockets and functional sites, retrieved from the CASTp 3.0 web server, http://sts.bioe.uic.edu/castp, Chicago, IL 60607, USA, accessed on 1 April 2023, are also shown in Figure 1. Based on the available data, the depicted are the amino acid sites to which inhibitors can interact.

### 3.2. JAK Inhibitors, Tofacitinib and Baricitinib, and Their Virtual Interaction with JAK Proteins

**Figure 2:**
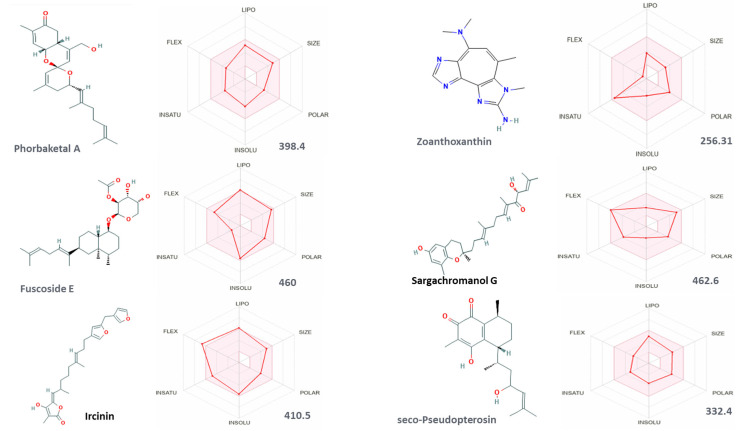
The structures of the proposed molecules as retrieved from the PubChem website. Representative images of some of the highlighted molecules for optimal behavioral, physiochemical properties, lipophilicity, water solubility, and pharmacokinetics characteristics, as analyzed by SwissADME software. Names and molecular weight in g/mol are listed.

After screening the active sites at JAK proteins, we evaluated the binding affinities and the docking score of tofacitinib and baricitinib to JAK1, JAK2, and JAK3, to be used as a reference to which our proposed inhibitors are compared. Molecular docking data indicated that tofacitinib and baricitinib can bind to the JAKs’ ATP-binding sites via conventional hydrogen (H-H) or carbon hydrogen (C-H) interactions. Virtual binding sites of tofacitinib and baricitinib to JAK proteins are shown in Table 1. In addition, the structures of the biomolecules as retrieved from the PubChem website are indicated in Figure 2 and Supplementary Figure S1.

#### 3.2.1. Tofacitinib and Baricitinib and Their Virtual Interaction with JAK1

Molecular docking analysis of ligands–protein interactions showed that tofacitinib can bind virtually by conventional hydrogen bond to JAK1 at ATP binding sites of the SH2 domain, including LYS908, LEU959, and GlY884, with binding energy of −7.8, −7.1, and −8 kJ/mol, respectively. Tofacitinib seems to have a robust H-H binding to another ATP binding residue, ARG1007, where the binding distance is 2 Å and the docking score (binding energy) is −7.1 kJ/mol. On the other hand, baricitinib interacts with JAK1 via H-H bonds at several ATP-binding sites, including LEU881, GLU883, HIS885, and PHE886, with binding energy between 7.1 and 7.9 kJ/mol. In addition, similarly to tofacitinib, a carbon hydrogen (C-H) interaction between baricitinib and JAK1 was seen at LYS908, binding energy −7.8. Moreover, baricitinib forms an H-H interaction with GLU957 (b.e. −7.9 kJ/mol), an amino acid at JAK1 essential for stabilizing the inhibitors. Together, these data reveal that the two FDA-approved molecules can effectively bind to the JAK1 ATP-binding sites, specifically LYS908, GLU957, and LEU959, and, thus, inhibit its phosphorylation and activity.

#### 3.2.2. Tofacitinib and Baricitinib and Their Virtual Interaction with JAK2

As shown in Figure 1, ATP-binding sites at JAK2 are located at amino acid sequences between (855–863) and at 882. Interestingly, we could not see an interaction between tofacitinib and baricitinib and JAK2 at any of these ATP-binding sites (Table 1). However, both can bind with hydrogen bond interaction to JAK2 at GLU930 and LEU932 (both with binding energy −7.8 kJ/mol), which seems to play an important role in the inhibition of JAK2 activity.

#### 3.2.3. Tofacitinib and Baricitinib and Their Virtual Interaction with JAK3

In the JAK3 protein, residues E903GLU903 and L905LEU905 of JAK3 can strongly stabilize the tofacitinib binding. In addition, ATP-binding sites were reported to be located at L828, V836, A853, V884, E903LEU828, VAL836, ALA853, VAL884, GLU903, and L905LEU905 CYS909 and L956LEU956 (Figure 1). Herein, we found that tofacitinib binds virtually to JAK3 at ARG948, TRY994, and ASP867 (b.e. −7 kJ/mol). However, tofacitinib and baricitinib can bind to JAK3 at EU905 (docking score −8.2 and 8.5 kJ/mol, respectively). 

### 3.3. Marine Biomolecules as Possible Inhibitors for JAK Proteins

**Figure 3:**
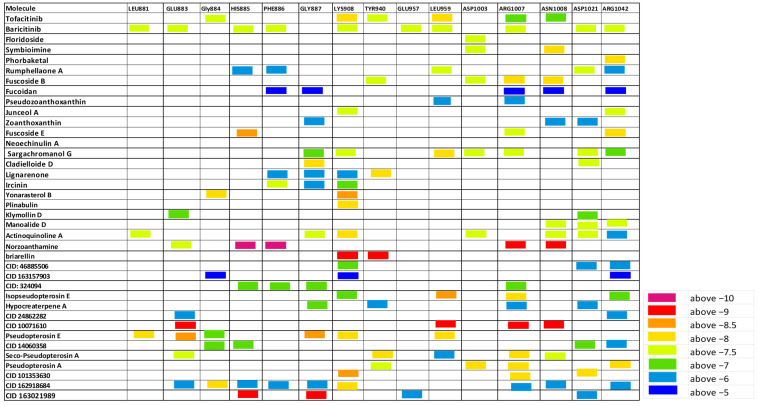
The estimated binding score of the marine biomolecules relative to JAK1 protein residues. A heat map is inserted to compare the binding affinities.

**Figure 4:**
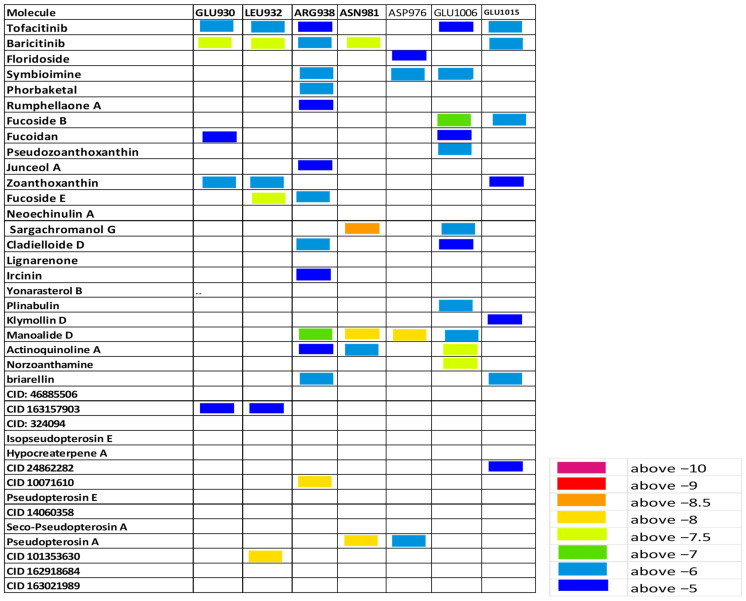
The estimated binding score of the marine biomolecules relative to JAK2 protein residues. A heat map is inserted to compare the binding affinities.

**Figure 5:**
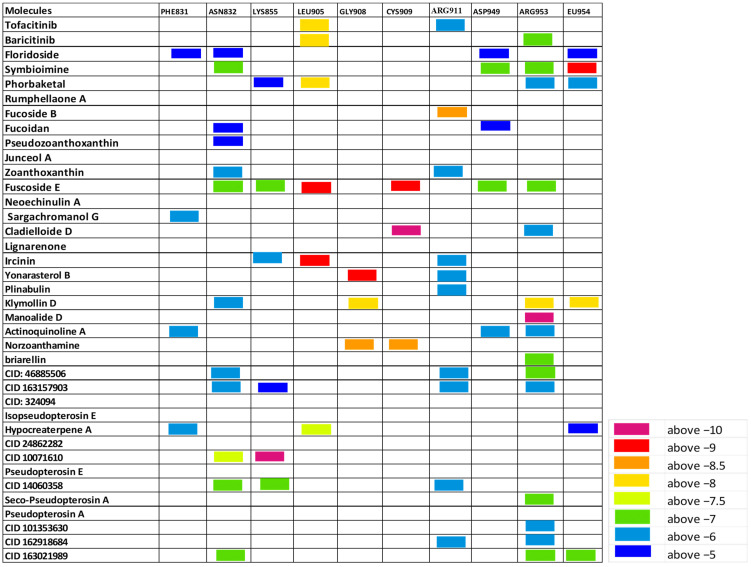
The estimated binding score of the marine biomolecules relative to JAK3 protein residues. A heat map is inserted to compare the binding affinities.

Herein, the binding affinity and sites of interaction of the listed molecules were compared to tofacitinib and baricitinib. A heat map based on the extent of binding energies (difference in color gradients) is shown in Figure 3, Figure 4 and Figure 5. The shortlisted active sites are LYS905, GLU957, LEU959, and ASP1003 at JAK1, GLU930 and LEU932 at JAK2, and GLU905 and CYS909 of JAK3.

#### 3.3.1. Marine Biomolecules as Potential Candidates Can Inhibit JAK1 Protein

**Figure 6:**
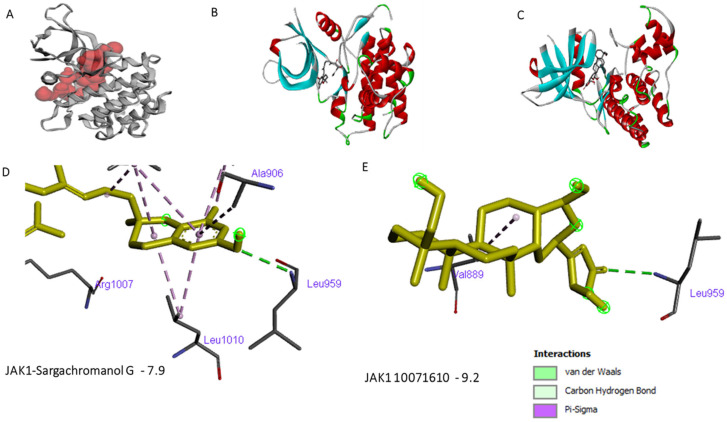
Representative images of the in silico binding of Sargachromanol G and CID 10071610 to JAK1 protein. (**A**). Details of the JAK1 binding sites as shown by the CASTp 3.0 web server. (**B**,**C**) 3D binding of the ligands to the depicted amino acids is shown, as analyzed by discovery studio software. (**D**,**E**) 3D details of the active residues binding. 2D images of more binding sites are shown in the Supplementary Materials.

Previous studies documented that several biomolecules from marine resources exhibit anti-inflammatory activity [15,16,20]; however, the possible inhibitory effect of many of these molecules on JAK proteins remains to be investigated. Our comparative docking and virtual analysis (Figure 3 and Figure 6) showed that out of more than 50 compounds tested for in silico interaction with JAK1, a set of molecules could be considered as potential candidates can be investigated in vitro and in vivo for their anti-inflammatory activity. These molecules include Sargachromanol G (SG), Isopseudopterosin E, Seco-Pseudopterosin A, and CID 10071610. In addition to interacting with the kinase active site of JAK1 and ASP1003, Sargachromanol binds mostly to the same JAK1 ATP binding sites to which tofacitinib and baricitinib are interacting (Figure 3, Supplementary Figure S2). In addition, the diterpenes, Isopseudopterosin E and Seco-Pseudopterosin A, bind to JAK1 active residues, LYS908 and LEU959, with a binding energy similar to that between the approved inhibitors and these residues. However, the compound CID 10071610 was found to interact with JAK1′ active sites, LEU959 and ASP1007, with binding energy of −9 kJ/mol, which is higher than that between the approved drugs and JAK1. Together, these results indicate that Sargachromanol G, Isopseudopterosin E, Seco-Pseudopterosin A, and CID 10071610 are potential candidate inhibitors of JAK1 to be investigated in vitro and in vivo for anti-inflammatory activity.

#### 3.3.2. Zoanthoxanthin and Fuscoside E May Inhibit JAK2 Activity

**Figure 7:**
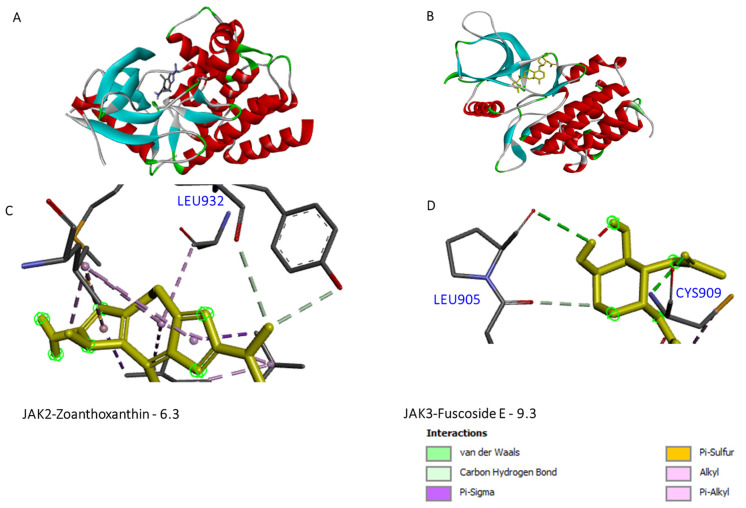
Representative images of the in silico binding of Zoanthoxanthin to JAK2 and -Phorbaketal A to JAK3. (**A**,**B**) Details of the JAK2 and JAK3 binding sites as shown by the CASTp 3.0 web server. (**C**,**D**) 3D binding of the ligands to the depicted amino acids is shown, as analyzed by discovery studio software; 2D images of more binding sites are shown in the Supplementary Materials.

Several recent studies pointed out that GLU930 and LEU932 of JAK2 are critical targets for hydrogen bonding interactions of inhibitors [1,21,22,23]. In this regard, both tofacitinib and baricitinib interact with these two active sites that are crucial for JAK2 activity (Figure 4 and Figure 7; Supplementary Figure S3). Similarly, the marine Zoanthoxanthin and compound CID 163157903 were found to interact virtually with these two active sites. In the meantime, Fuscoside E and molecule CID 101353630 were shown to have higher binding affinity to LEU932 relative to tofacitinib. Together, these data indicate that marine compounds such as Zoanthoxanthin and Fuscoside E may inhibit JAK2 activity when being investigated preclinically or clinically.

#### 3.3.3. Phorbaketal and Fuscoside E Are Potential Candidates That May Inhibit JAK3 Activity

At JAK3, there are three critical sites, CYS909, LYS905, and GLU908, required for its activity and for the inhibitor stability. Our data showed that Phorbaketal, Fuscoside E, and Ircinin can virtually bind to JAK3 critical residues, including LYS855 and LEU905, with a binding energy higher than those between the approved inhibitors and JAK3 (Figure 5 and Figure 7; Supplementary Figure S3). In addition, Norzoanthamine interacts with important cites at JAK3, GLU908, and CYS909. However, Fuscoside E interacts with other active sites at JAK3, such as ASP867 and ARG911, that are critical for JAK3 activity. Together, these data indicate that Phorbaketal and Fuscoside E and are good candidates that may inhibit JAK3 activity.

### 3.4. Analysis of MD Simulations Outcomes

**Figure 8:**
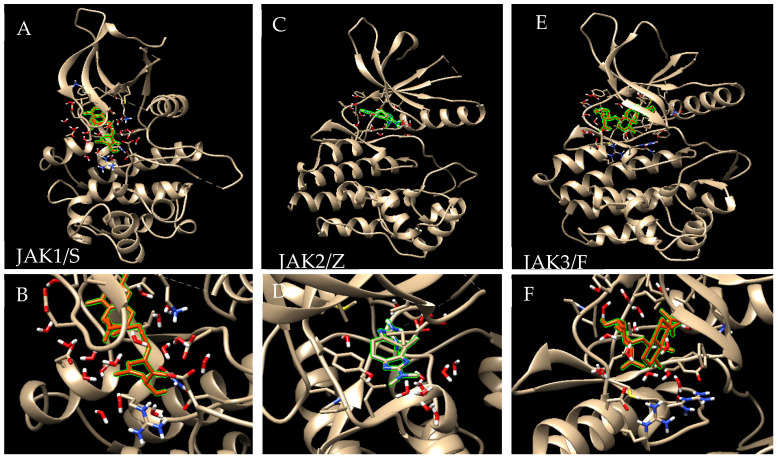
MD simulation to validate the molecular docking results. Shown are the kinases/ligand complexes including (**A**,**B**) JAK1(6sm8)/SG, (**C**,**D**) JAK2 (3jy9)/ZAX, and (**E**,**F**) JAK3 (6pjc)/Fuscoside E.

**Figure 9:**
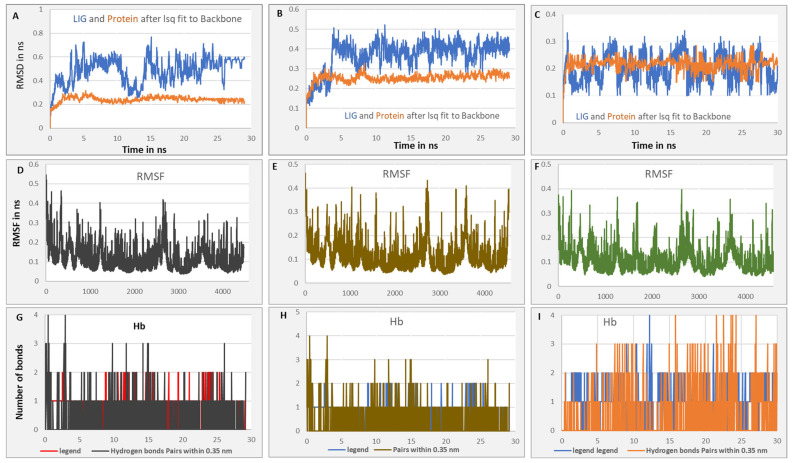
The average RMSD with time is lower than 4 Å (**A**–**C**). The RMSF values by time during MD simulation (**D**–**F**). H-bonds formation (**G**–**I**).

To this end, our comparative molecular dynamics analysis suggested a set of marine molecules, and then we used MD simulation (for 30 ns) to validate the molecular docking results through exposing the most promising kinases/ligand complexes, including JAK1(6sm8)/SG, JAK2 (3jy9)/ZAX, and JAK3 (6pjc)/Fuscoside E (Figure 8 and Figure 9), where stable and lower binding energy were found based on analyzing the set of parameters. These parameters included root-mean-square deviation (RMSD) and root-mean-square fluctuation (RMSF).

During MD simulation, the average RMSD with time is lower than 4 Å (between 0.2 and 0.4 nm, Figure 9A–C). RMSD analysis reflecting optimal distances between atoms (i.e., ligand, protein, and backbone atoms) shows acceptable values suggesting stable binding. In the meantime, the RMSF, which estimates the average deviation or particles fluctuation (e.g., a protein residue), showed minimal fluctuations by time during MD simulation (less than 4 Å for the tested complexes, Figure 9D–F), suggesting a specific and a stable interaction. Additionally, the interaction and the potential and the total energy between the three kinases and the tested inhibitors appear to be very low, suggesting a stable and a higher binding affinity (Table 2). Moreover, we analyzed the atoms overlap between the kinases and the ligands, through calculating hydrogen bonds formation (Figure 9G–I and Supplementary Materials). For the JAK1/SG complex, the overlap between JAK1 (4486 atoms) and SG (69 atoms) showed that there are 403 donors and 799 acceptors. However, for the JAK2/ZTX complex, hydrogen bonds between JAK2 (4661 atoms) and ZAX (37 atoms) indicated 425 donors and 843 acceptors. Additionally, for JAK3/FU, there were hydrogen bonds between JAK3 (4550 atoms) and FU (77 atoms), 421 donors and 824 acceptors. For more information about the complex’s behavior, please see the attached videos.

## 4. Discussion

In the current study, more than 200 nontoxic marine compounds having antioxidant or anti-inflammatory properties were selected to be screened and tested for optimal physiochemical properties, pharmacokinetics, and drug-likeness characteristics. Next, structural investigation and molecular docking analysis were conducted to explore the possible inhibitory effects of the selected molecules, those with optimal properties, on JAKs proteins. Heat maps based on the binding affinities of at least 35 molecules to JAKs protein-critical residues are shown in Figure 3, Figure 4 and Figure 5.

Therapeutic inhibitors include anti-inflammatory and analgesic drugs, glucocorticoids, immunosuppressive agents, and DMARDs. Of these drugs, the inhibitors of JAKs are the latest class of DMARDs to emerge in treating RA [8,9]. To inhibit JAK enzymes, the recently approved and/or the developed drugs aimed to obstruct ATP binding. The pseudokinase domain of JAKs is a major regulatory domain for the kinase activity [8]. JAK1 is a widely expressed enzyme in human tissues that can be phosphorylated by four cytokine-receptor families and, in the meantime, can phosphorylate all STATs to enhance inflammatory mechanisms [4]. Residues GLU957 and LEU959, located at the JAK1 hinge region, are among the JAK1 ATP-binding sites that were reported to be the most selective residues for the H-bond interaction, critical for selective inhibition of JAK1, and essential for stabilizing the inhibitors [24,25]. In the meantime, another ATP-binding site, LYS908, is one of the JAK1 residues that mediate JAK1 major functions [26,27]. Herein, we found that baricitinib interacts with JAK1 at GLU957 with high binding energy (−7.9 kJ/mol). However, both tofacitinib and baricitinib interact with JAK1 at LEU959 through H-H and H-C binding, respectively, with a bind score of −7.8. In agreement with this finding, Sargachromanol was found to interact with JAK1 active/ATP binding sites at the SH2 domain, including LYS908, LEU959, and ARG1007, with the binding affinities and energy similar to those of the approved inhibitors, tofacitinib and baricitinib. Additionally, Sargachromanol interacts with the critical active residues of JAK1 and ASP1003, which is not the case for tofacitinib and baricitinib. In consistency with our finding, Sargachromanol isolated from the brown alga Sargassum siliquastrum was reported as an anti-inflammatory molecule, where it inhibited osteoclastogenesis by suppressing the activation MAPKs and NF-κB in inflammatory cell model [28,29]. Sargachromanol was also found to regulate the expression of osteoclastogenic factors in human osteoblast-like cells [30]. Pseudopterosin and Isopseudopterosin belong to diterpene marine natural products, which have been reported to have effective anti-inflammatory and analgesic properties [31]. However, their effects on RA have not been described. These pseudopetrosins were found to have a similar binding affinity to JAK1 LEU908 and LEU959, relative to the binding affinities of tofacitinib and baricitinib to these JAK1 active sites. Since the selectivity of an inhibitor like Upadacitinib toward the JAK1 was raised mainly due to the higher total internal and binding energy [24], the higher docking score seen between Sargachromanol, Isopseudopterosin E, Seco-Pseudopterosin A. and CID 10071610, and JAK1′ LEU959, suggests a higher stability at the binding site. In the present study, tofacitinib showed H-H binding affinity to JAK1′ LYS908; however, baricitinib displayed C-H interaction, and the binding energy was −7 and −7.8 kJ/mol, respectively. Interestingly, Sargachromanol and Pseudopterosin showed higher binding energy to LYS908 compared to the two inhibitors. Other important JAK1-ATP-binding sites were found to be potential targets for several recently developed inhibitors (listed in Figure 1), including LEU881, ASN1008, and ASP1021 [11,28]. Of these residues, tofacitinib and Pseudopterosin are interacting with JAK1 at LEU881; Fuscoside E, tofacitinib, and other molecules bind to ARG1008; and Fusco-side E, baricitinib, and Sargachromanol bind to ASN1007. The negative feedback regulators of the JAK-STAT, suppressors of cytokine signaling (SOCS), were reported to inhibit the activity of JAK kinase, where ASP1042 and ASP1040 of JAK1 were shown to be key components and the targets of SOCS. In this regard, most of the current highlighted JAK1 possible inhibitors, Sargachromanol, Pseudopterosins, CID 10071610, and Fuscoside E bind to JAK1 protein ASP1042 residue through H-H interaction, with binding energy of −7.5 and −9.2 kJ/mol. 

According to several recent studies, GLU930 and LEU932 of JAK2 were found to be a target for hydrogen bonding interactions of inhibitors [13,21,23]. The amino acid residues LEU855, GLY858, ARG980, and LEU983 are also involved in the formation of the JAK2–ligand complex [32]. Our data showed that tofacitinib binds virtually to JAK2 at GLU930 (through H-H binding) and at LEU932 (through H-H and C-H binding), both with binding energy of −6.9. In the meantime, baricitinib binds to the two residues through H-H interaction, with binding energy of −7.8 kJ/mol. However, interestingly, molecules such as ATX and PFFE (with molecular weight above 480) were able to form H-H bonds with JAK2 at GLU930 with higher binding energy (−8.1 and −9.8 kJ/mol, respectively) compared to tofacitinib and baricitinib. Additionally, Zoanthoxanthin and compound CID 163157903 showed a binding affinity above −5.5 kJ/mol. In the meantime, Fuscoside E forms H-H bond with JAK2 at that residue (b.e. −7.8 kJ/mol). In addition to the residues GLU930 and LEU932, the JAK2 binding site was suggested to contain other important residues, such as ASP994, LEU855, LEU983, ARG980, and PHE995, that are critical for JAK2 activity [32]. 

The molecular dynamics simulation suggested that the residues GLU903 and LEU905 of JAK3 strongly stabilize the tofacitinib binding. In addition, in the JAK3 protein, CYS909 serves as a potential target for the covalent attachment of inhibitors [33]. In agreement with that, we recently showed that Fuscoside E can bid to two key regulators residues of JAK3, required for its activity and for the inhibitor stability, CYS909 and LYS905, with a binding energy (−9.6 kJ/mol) higher than tofacitinib [34]. Additionally, other important targets of the inhibitors at JAK3 are residues ARG911 and TYR994, to which some of the present investigated molecules virtually interacted. 

Based on the current in silico data, Zoanthoxanthin (265 gm/mol), Fuscoside E (460 gm/mol), and CID 163157903 (66) interact with JAK2 active residues, GLU930 and LEU932. However, Phorbaketal, Fuscoside E, Ircinin, Norzoanthamine, and Hypocreaterpene A (84) interact with at least two sites of JAK3 active sites, including LYS855, LEU905, and GLY908. Classical studies indicate that Fuscoside E (FsE) is among the anti-inflammatory diterpenoids that can be extracted from marine resources [34]. In parallel with that, we recently proposed Fuscoside E as a potential inhibitor of JAK3 [35]. Phorbaketal A (mw around 400 g/mol) is a tricyclic sesterterpenoid isolated from the marine sponge with little information available about its biological activities. Related to that, Phorbaketal A, isolated from the marine sponges, inhibited the production of inflammatory mediators through retarding the NF-κB pathway and promoting the HO-1 pathway [15]. The negative total energy in MD simulation steps in the current model means that energy reaches its minimum values and the system is stable. The values of RMSD and RMSF also supported a stable and specific binding between the highlighted molecules and the JAK proteins. Although more attention has been recently given to finding new drugs that can potentially relieve RA conditions [4,5,6,7,11,36], the recognition of selected antirheumatoid arthritis through the inhibition of Janus kinases signaling still in need. 

## 5. Conclusions

We provide a detailed analysis for the amino acid residues at JAK proteins to which the currently proposed set of bioactive molecules are interacting. Our reference was the comparative analysis of the FDA-approved inhibitors tofacitinib and baricitinib. We propose a potential inhibitory effect of Fuscoside E Sargachromanol G, Zoanthoxanthin, Phorbaketal, and Pseudopterosins on JAK proteins. A promising role of these marine bioactive molecules can be confirmed in prospective preclinical/clinical investigations using rheumatoid arthritis models.

## Figures and Tables

**Table 1:** Tofacitinib and baricitinib and their virtual interaction sites and binding score to JAK proteins.

Bond Interaction (Energy in kJ/mol)	Docking Score	Bound Amino Acids Residues	Distance in Angstrom (A)	Docking Score	Bound Amino Acids Residues	Distance in Angstrom (A)
Tofacitinib Virtual binding to JAK1				Baricitinib Virtual binding to JAK1		
Conventional Hydrogen	−7.8	SER996, ARG997	3.15, 3.31	−7.1	ARG930	3.12
Hydrogen Carbon		TYR940	3.33		SER996	3.24
Conventional Hydrogen	−7.6	Gly884	3.22	−7.9	GLU957, GLU883	2.48, 3.09
Hydrogen Carbon		GLY1023	3.5		LEU881	3.31
Conventional Hydrogen	−7.1	ARG1007, ASN1008	1.97, 2.79	−7.8	HIS885, HIS918, ARG1007, HIS918, PHE886, ARG1007, LYS905	2.99, 3.18, 2.20, 3.18, 3.2, 2.2, 2.94
Hydrogen Carbon		LYS908	3.5		LYS908, LUE959, ASP1021, ARG1042	3.10, 2.94, 3, 3.3
Conventional Hydrogen	−8.2	LEU959	2.57			
tofacitinib Virtual binding to JAK2				Baricitinib Virtual binding to JAK2		
Conventional Hydrogen	−6.9	GLU930, LEU932	2.88, 3.37	−7.8	GLU930, LEU932	2.18, 3.01
Hydrogen Carbon		LEU932	3.30		ASN981, LEU932	3.76, 3.47
Conventional Hydrogen	−5.8	ARG938	3.1	−6	ARG938, SER1056	3.28, 2.98
Hydrogen Carbon		GLU1015	3.65		GLU1015	3.58
Conventional Hydrogen	−6.2	VAL1033, ALA1034	3.25, 3.98	−5.4	ARG1090, ASP1092, TRY1099	2.98, 3.19, 2.98
Hydrogen Carbon		GLU1006	3.48		ARG1090, PRO1091	3.45, 3.62
tofacitinib Virtual binding to JAK3				Baricitinib Virtual binding to JAK3		
Conventional Hydrogen	−7	ARG948, ASP867, TRY994	2.99, 2.54, 2.92	−7.4	GLN915, ARG953, ASN954, CYS1024	3.32, 3.13, 1.98,
Conventional Hydrogen	−8.2	LEU905	2.25	−8.5	LEU905	2.06
Conventional Hydrogen	−6.9	GLN988, ARG911	2.8, 3.16	−5.5	ALA923, GLU1069, TRY1023	2.06, 2.25, 2.92,
Conventional Hydrogen	−5.6	GLU1069, ALA923, TYR1023	2.20, 2.17, 2.83, 2.20	−6.8	ARG948, SER989, TRY994	3.03, 3.23, 3.01
Hydrogen Carbon		GLU1098	2.8		GLU985, PRO986,	3.49, 3.39

**Table 2:** The interaction and the potential and the total energy between the three kinases and the tested inhibitors, showing energy and total energy drift (Err. Est. and Tot-drift, respectively).

Energy (kJ/mol)	Average	Err. Est.	RMSD	Tot-Drift
JAK1/SG				
bond	3631.04	1.1	100.266	−6.71359
Angle	168.444	0.58	19.5854	1.21744
LJ (SR)	63,434.2	4.9	722.14	−25.6262
Potential	−507907	48	809.057	−326.584
Kinetic En.	100067	2.6	564.691	−6.83606
Total Energy	−407840	50	1075.7	−333.421
JAK2/ZAX				
bond	3773.51	0.85	101.737	4.76318
Angle	145.789	2.5	16.249	−13.6934
LJ (SR)	58,140.2	21	694.993	133.869
Potential	−482785	42	789.258	−238.977
Kinetic En.	94501	5.9	553.496	−30.7843
Total Energy	−388284	47	1056.88	−269.758
JAK3FU				
bond	3790.28	1.7	102.87	−0.10374
Angle	188.253	0.63	20.8998	−3.61546
LJ (SR)	64,021.7	6.7	725.951	28.1077
Potential	101552	3.6	570.884	−7.54866
Kinetic En.	−518637	21	813.684	−145.281
Total Energy	−417085	22	1086.43	−152.829

## Data Availability

The data presented in this study are available on request from the corresponding author.

## Supplementary Materials

The following supporting information can be downloaded at: https://www.mdpi.com/article/10.3390/cimb46090631/s1, Figure S1: Representative images of some the highlighted molecules for optimal behavioral, physiochemical properties, lipophilicity, water solubility, and pharmacokinetics characteristics, as analyzed by SwissADME software representative images; Figure S2 & Figure S3: 2D images of the interactive sites with JAKs. Video S1: MD simulation videos of the three shortlisted molecules with JAKS.
