# Supplementary material for: Marine Bioactive Molecules as Inhibitors of the Janus Kinases: A Comparative Molecular Docking and Molecular Dynamics Simulation Approach
Source: Curr Issues Mol Biol. 2024 Sep 23;46(9):10635–50. doi: 10.3390/cimb46090631 (PMC11430628; doi:10.3390/cimb46090631)
Supplement: Supplementary file 1 [file cimb-46-00631-s001.zip › cimb-3202556-supplementary.pdf]

Supplementary Figures

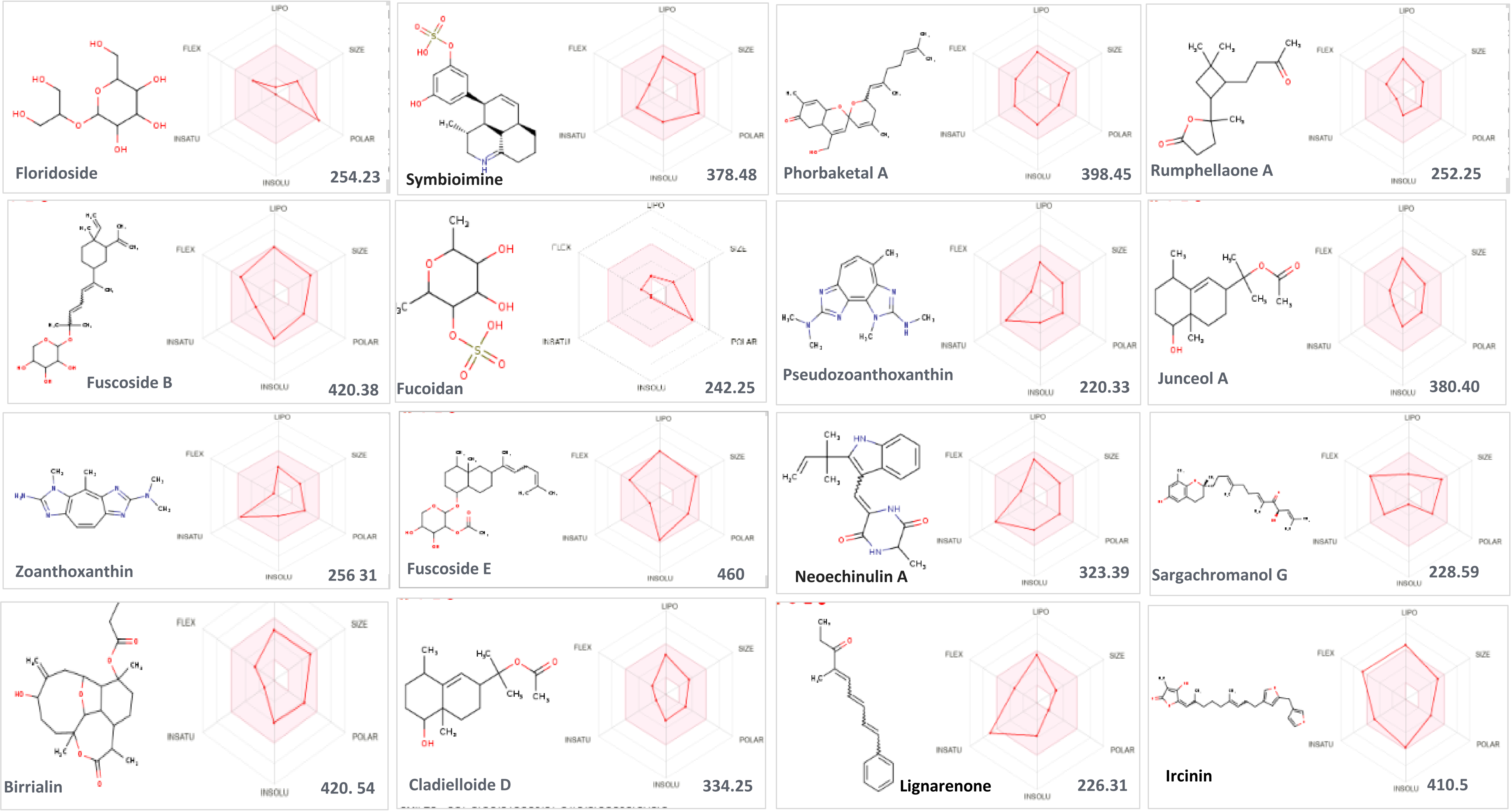

Suppl Figure 1.

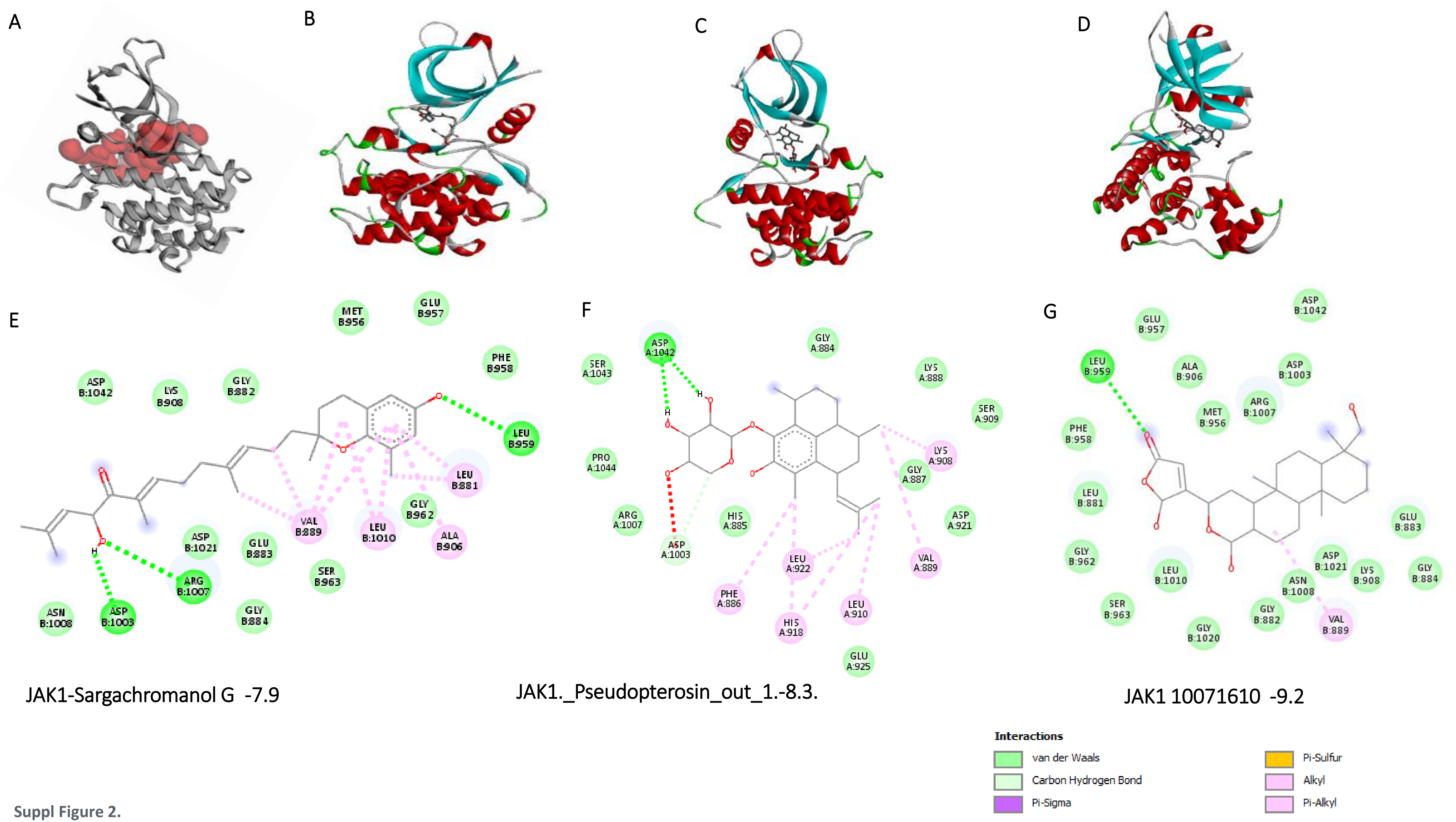

Suppl Figure 2.

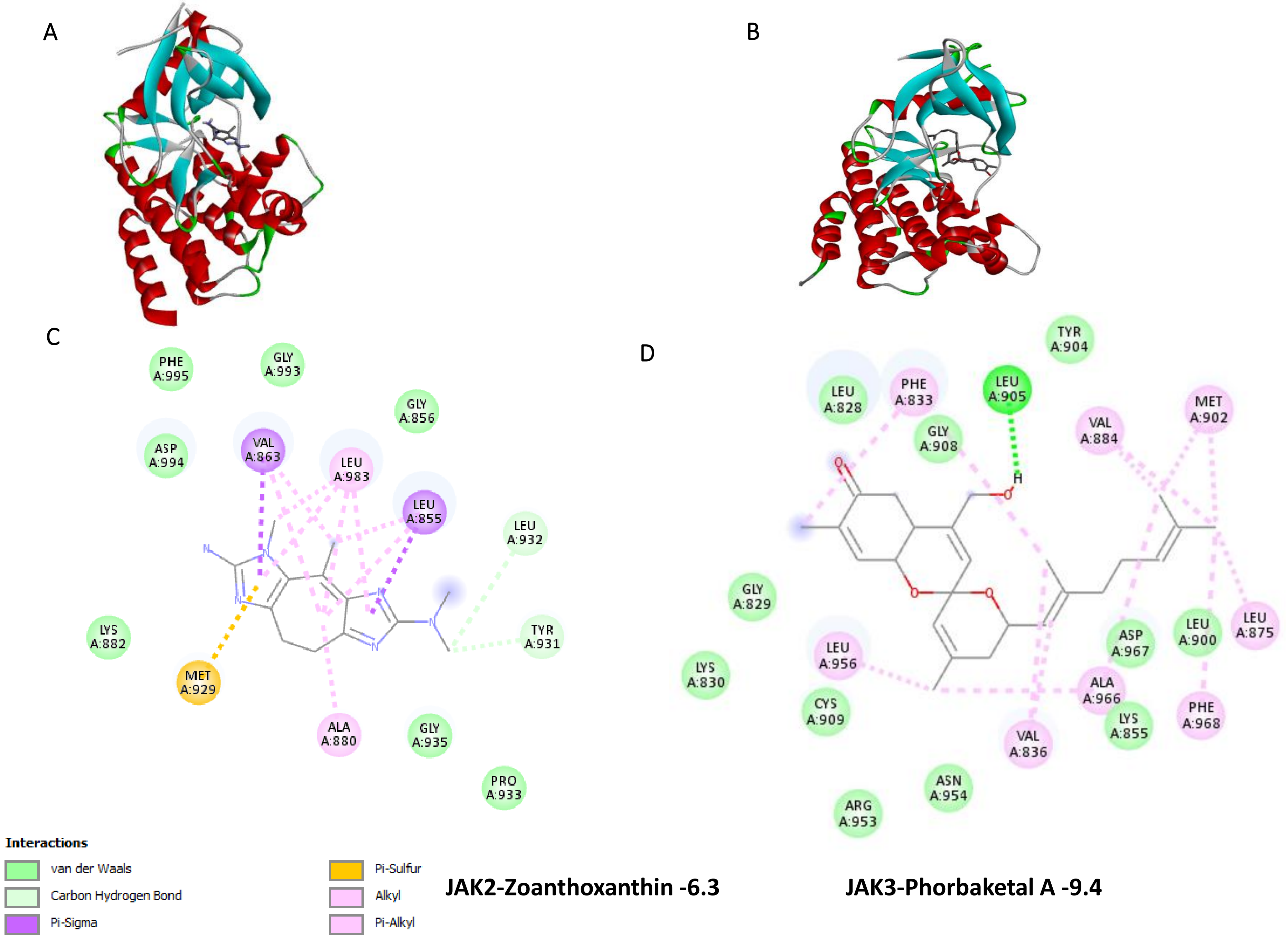

JAK2-Zoanthoxanthin -6.3

JAK3-Phorbaketal A -9.4

Suppl Figure 3.

Supplementary materials 2

2.2.

- Preparation of 3D protein structure (from the Protein Data Bank, https://www.rcsb.org) as seen by discovery studio software, the shown retrieved Kinase is 3jy9 (JAK2).

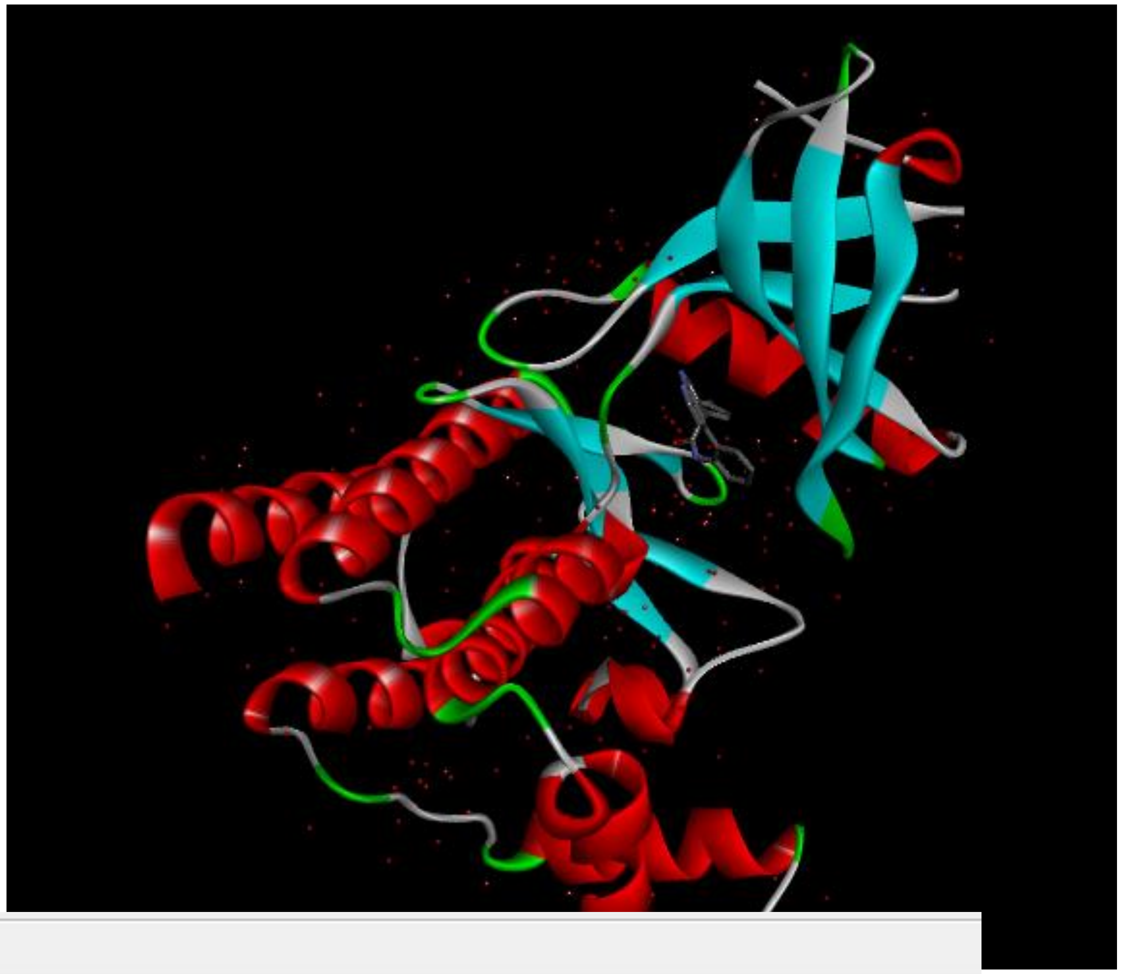

- Below is the retrieved legends protein complexes as prepared and purified using discovery studio steps legend purification, water removal and heteroatom deletion

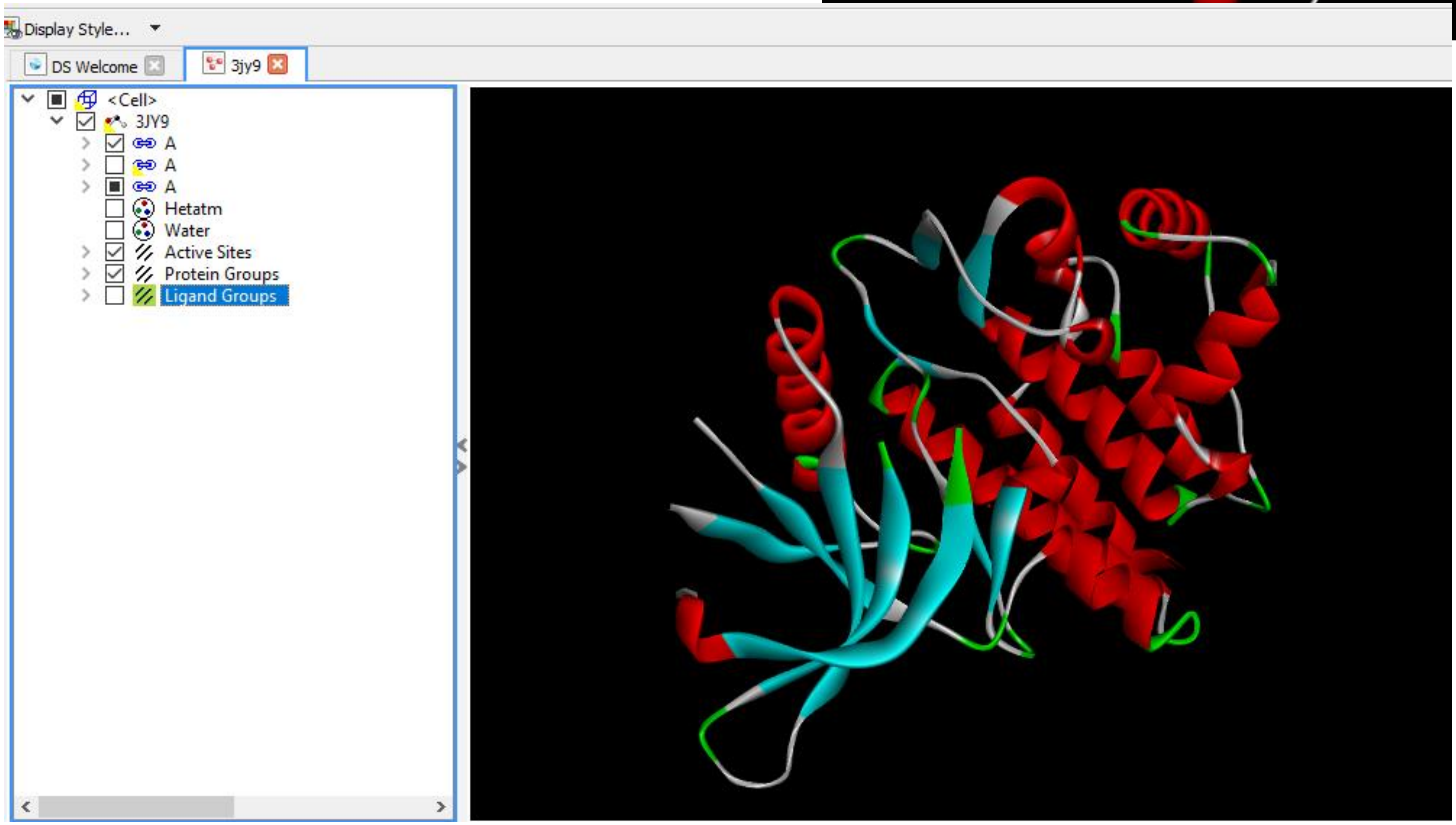

2.2. Preparation of 3D protein structure

- **The** retrieved legends protein complexes as prepared and purified from heteroatom and coordinates

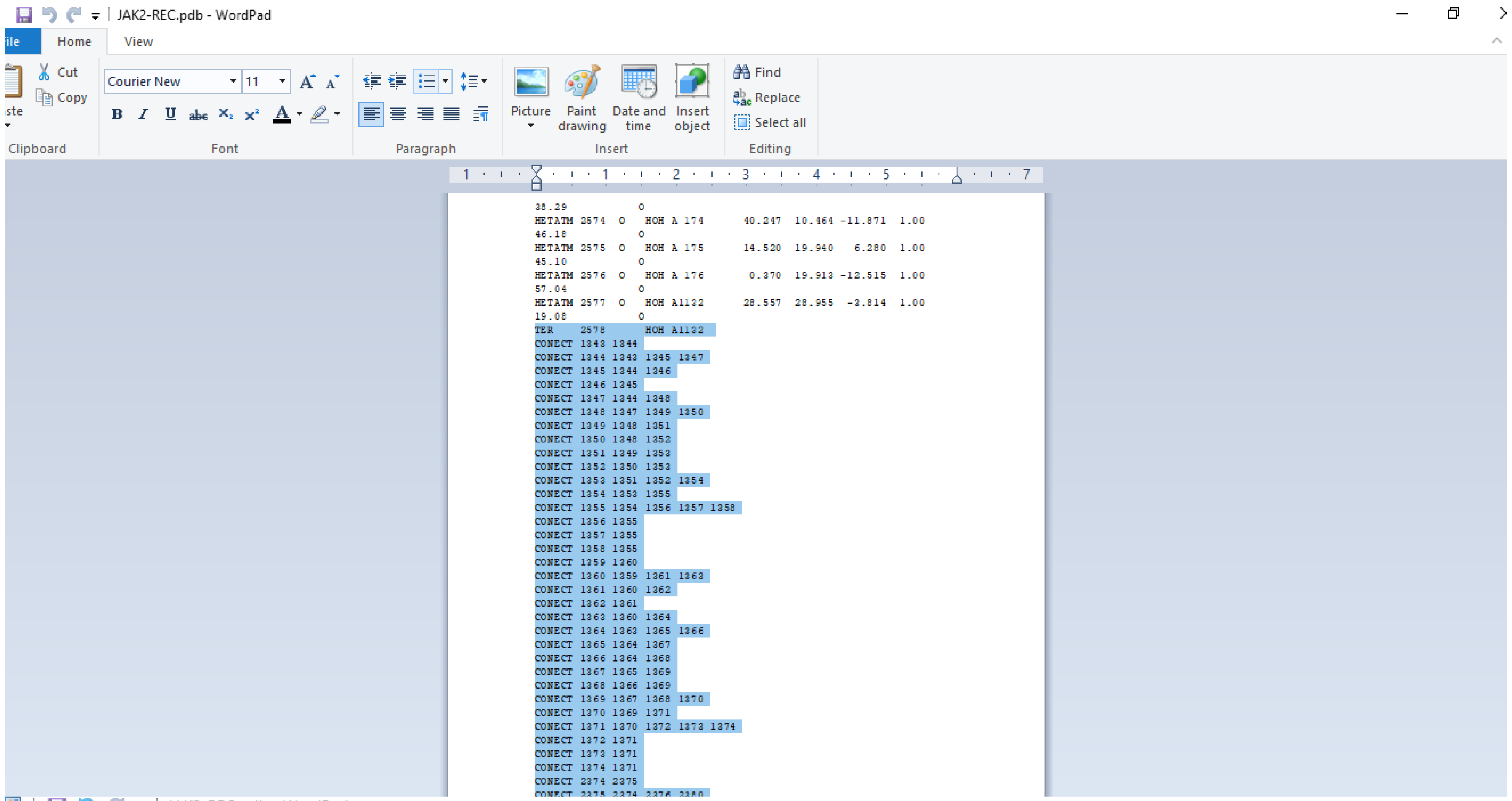

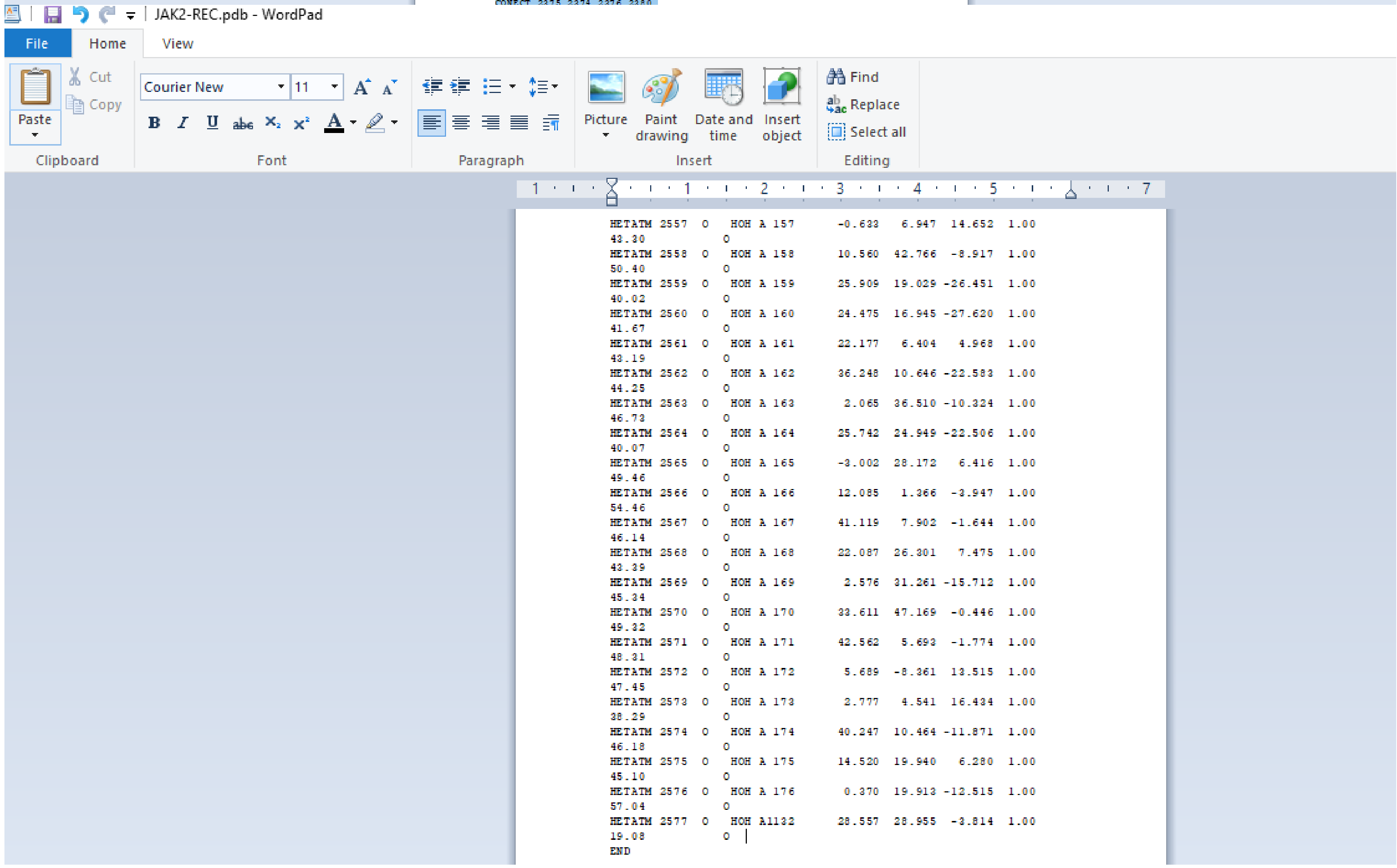

2.2. Preparation of 3D protein structure

For further purification of the retrieved protein complexes, the prepared receptor (kinase) was and purified using the Swiss pdb viewer software. Details of the legend purification and deletion and energy minimization steps are shown below and in the nest slides

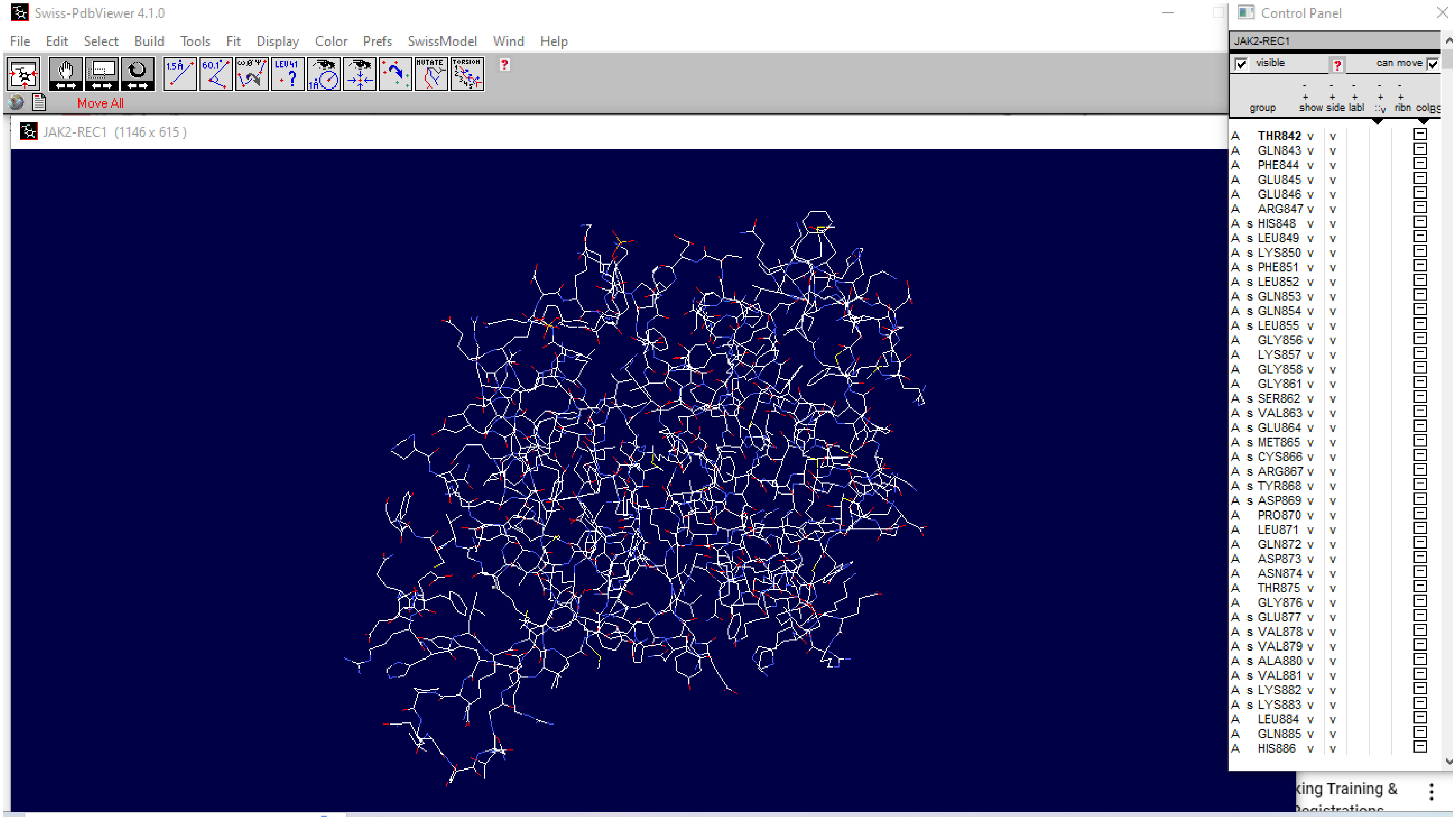

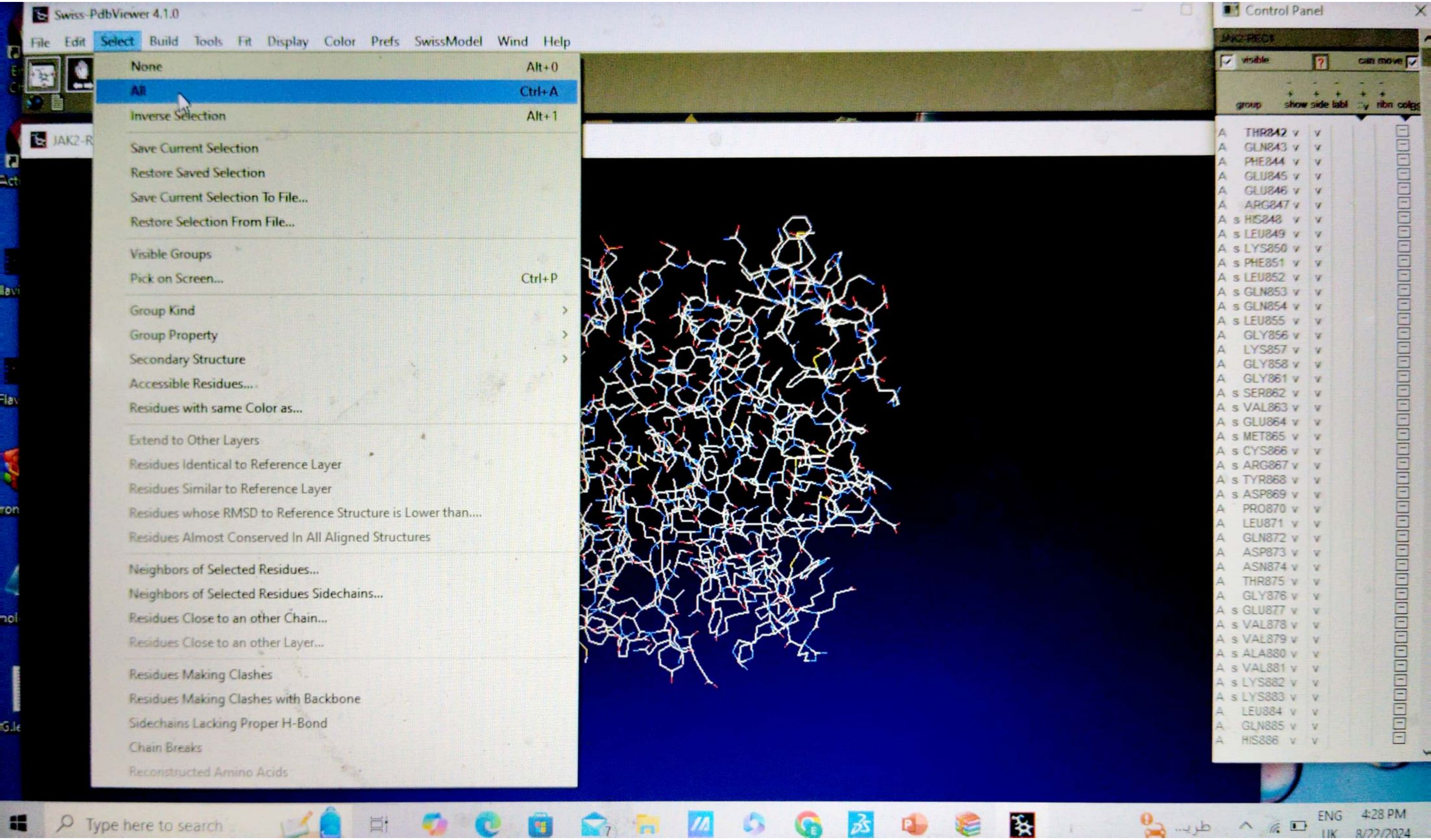
Swiss-PdbViewer 4.1.0
File Edit Select Build Tools Fit Display Color Prefs SwissModel Wind Help
None Alt+0
All Ctrl+A
Inverse Selection Alt+1
Save Current Selection
Restore Saved Selection
Save Current Selection To File...
Restore Selection From File...
Visible Groups
Pick on Screen... Ctrl+P
Group Kind
Group Property
Secondary Structure
Accessible Residues...
Residues with same Color as...
Extend to Other Layers
Residues Identical to Reference Layer
Residues Similar to Reference Layer
Residues whose RMSD to Reference Structure is Lower than....
Residues Almost Conserved In All Aligned Structures
Neighbors of Selected Residues...
Neighbors of Selected Residues Sidechains...
Residues Close to an other Chain...
Residues Close to an other Layer...
Residues Making Clashes
Residues Making Clashes with Backbone
Sidechains Lacking Proper H-Bond
Chain Breaks
Reconstructed Amino Acids
Control Panel
visible
can move
group
show side labl
ribn
THR842
GLN843
PHE844
GLU845
GLU846
ARG847
HIS848
LEU849
LYS850
PHE851
LEU852
GLN853
GLN854
LEU855
GLY856
LYS857
GLY858
GLY861
SER862
VAL863
GLU864
MET865
CYS866
ARG867
TYR868
ASP869
PRO870
LEU871
GLN872
ASP873
ASN874
THR875
GLY876
GLU877
VAL878
VAL879
ALA880
VAL881
LYS882
LYS883
LEU884
GLN885
HIS886
Type here to search
ENG
4:28 PM

CamScanner

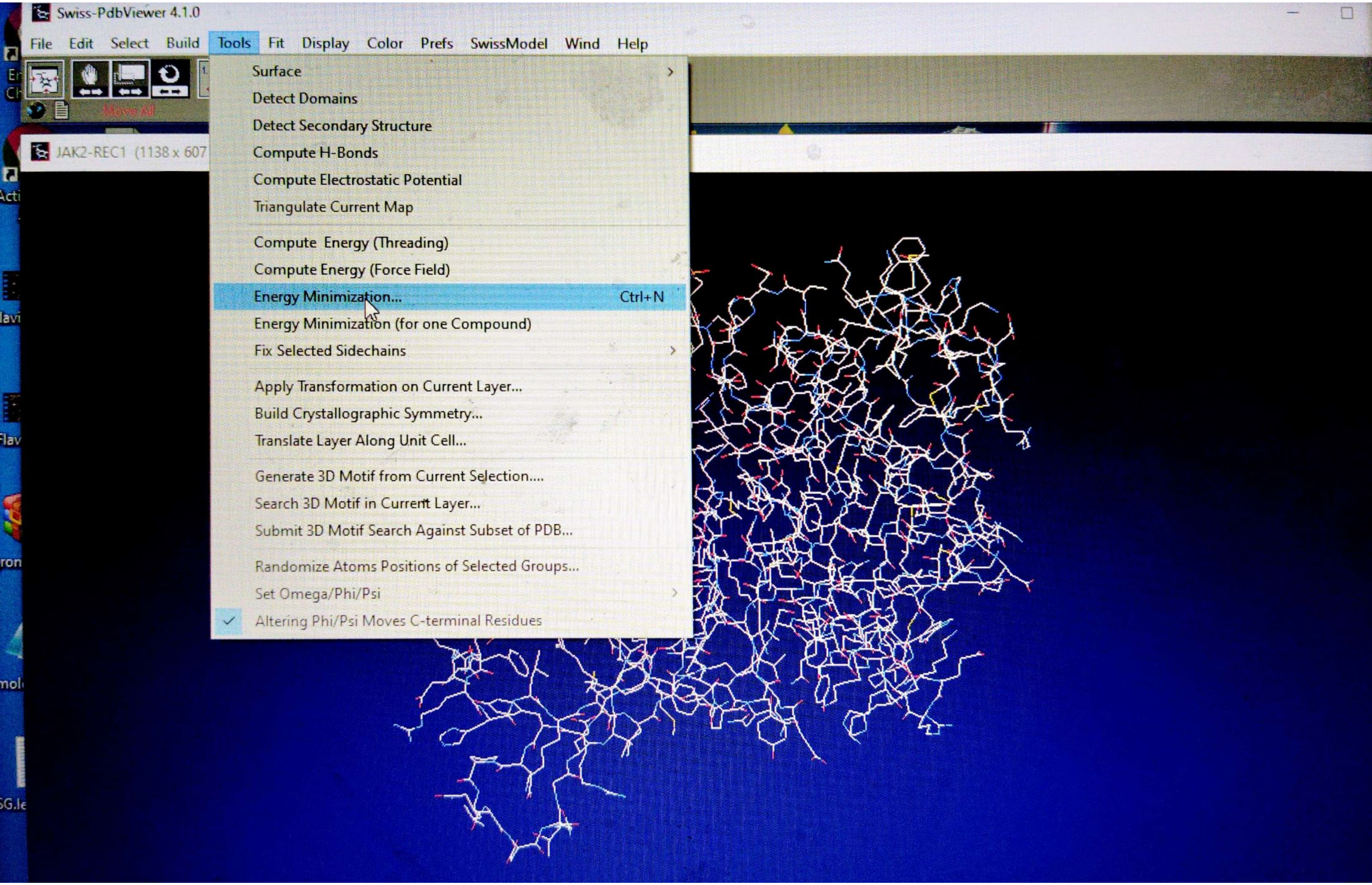
Swiss-PdbViewer 4.1.0
File
Edit
Select
Build
Tools
Fit
Display
Color
Prefs
SwissModel
Wind
Help
JAK2-REC1 (1138 x 607
Surface
Detect Domains
Detect Secondary Structure
Compute H-Bonds
Compute Electrostatic Potential
Triangulate Current Map
Compute Energy (Threading)
Compute Energy (Force Field)
Energy Minimization... Ctrl+N
Energy Minimization (for one Compound)
Fix Selected Sidechains
Apply Transformation on Current Layer...
Build Crystallographic Symmetry...
Translate Layer Along Unit Cell...
Generate 3D Motif from Current Selection....
Search 3D Motif in Current Layer...
Submit 3D Motif Search Against Subset of PDB...
Randomize Atoms Positions of Selected Groups...
Set Omega/Phi/Psi
Altering Phi/Psi Moves C-terminal Residues

CamScanner

Swiss-PdbViewer 4.1.0

File Edit Select Build Tools Fit Display Color Prefs SwissModel Wind Help

Move All

JAK2-REC1 (1138 x 607)

C:\Users\emad2\AppData\Local\Temp\Rar$EX30.593\SPDBV_4.10_PC\temp\energy.E22

/ Computations were done in vacuo with the GROMOS96 43B1 parameters set, without reaction field.
/ For more information about GROMOS96, refer to: W.F. van Gunsteren et al. (1996) in Biomolecular
/ simulation: the GROMOS96 manual and user guide. Vdf Hochschulverlag ETHZ (http://igc.ethz.ch/gromos).
/ When using those results, please mention that energy computations were done with the GROMOS96
/ implementation of Swiss-PdbViewer.

/ residue	bonds	angles	torsion	improper	nonBonded	electrostatic	constraint //	TOTAL
HHT A 842	0.160	2.910	7.483	0.000	0.00	24.41	0.0000 // E=	34.961
THR A 842	3.580	5.572	6.162	2.964	-8.44	148.91	0.0000 // E=	158.751
GLN A 843	1.336	7.744	6.170	0.574	-21.72	-148.26	0.0000 // E=	-154.157
PHE A 844	0.152	1.120	5.502	0.532	-54.34	0.94	0.0000 // E=	-46.096
GLU A 845	0.265	2.242	6.100	3.010	-33.70	-8.12	0.0000 // E=	-30.204
GLU A 846	0.384	2.745	9.568	0.735	-32.78	-7.04	0.0000 // E=	-26.389
ARG A 847	1.374	7.182	5.144	1.754	-20.96	-246.05	0.0000 // E=	-251.557
HISA A 848	0.318	3.710	4.455	0.252	-47.23	6.56	0.0000 // E=	-31.927
LEU A 849	0.148	7.999	13.450	1.560	-40.11	-8.54	0.0000 // E=	-25.499
LYSH A 850	0.368	1.744	12.824	0.918	-36.91	-18.92	0.0000 // E=	-39.970
PHE A 851	0.291	0.863	2.651	0.514	-41.73	11.21	0.0000 // E=	-26.204
LEU A 852	0.397	4.947	2.079	1.517	-38.00	11.60	0.0000 // E=	-17.469
GLN A 853	1.150	3.435	6.423	0.395	-25.68	-176.36	0.0000 // E=	-190.632
GLN A 854	1.056	3.690	9.516	0.462	-30.59	-158.25	0.0000 // E=	-174.112
LEU A 855	0.976	6.410	2.452	1.354	-33.73	42.16	0.0000 // E=	19.624
GLY A 856	0.407	1.062	0.857	0.541	-11.38	26.10	0.0000 // E=	17.580
LYSH A 857	1.075	3.432	16.131	0.139	-15.25	33.99	0.0000 // E=	39.515
GLY A 858	0.970	2.553	2.398	0.202	-5.54	145.15	0.0000 // E=	145.729
OXT A 858	0.000	0.000	0.000	0.000	15.81	-45.05	0.0000 // E=	-29.243
HHT A 861	3.190	14.316	7.205	0.000	0.00	41.71	0.0000 // E=	66.421
GLY A 861	0.659	1.605	1.817	0.198	3.17	81.37	0.0000 // E=	88.823
SER A 862	0.319	2.270	5.611	0.422	-28.35	-22.35	0.0000 // E=	-42.081
VAL A 863	0.688	2.236	1.974	1.550	-29.60	-19.15	0.0000 // E=	-42.295
GLU A 864	0.523	2.567	1.286	0.467	-44.81	-24.00	0.0000 // E=	-63.967
MET A 865	0.433	1.380	10.106	0.546	-43.72	-21.75	0.0000 // E=	-53.007
CYSH A 866	0.184	0.545	6.593	0.135	-42.21	-17.99	0.0000 // E=	-52.752
ARG A 867	0.953	3.276	7.145	0.834	-52.70	-271.88	0.0000 // E=	-312.370
TYR A 868	0.312	1.624	5.943	0.876	-59.37	-72.19	0.0000 // E=	-122.809
ASP A 869	1.120	4.806	6.540	1.239	-37.86	10.09	0.0000 // E=	-14.057
PRO A 870	0.400	14.343	17.174	0.815	-27.98	-12.81	0.0000 // E=	-8.068
LEU A 871	1.635	10.339	9.708	0.991	-20.66	8.65	0.0000 // E=	10.662
GLN A 872	2.346	10.176	1.987	2.398	-24.50	-159.49	0.0000 // E=	-167.079
ASP A 873	1.567	4.146	2.911	1.675	-16.06	-2.92	0.0000 // E=	-8.681
ASN A 874	1.470	8.603	0.791	0.935	-20.03	-167.88	0.0000 // E=	-176.105
THR A 875	1.106	1.639	4.524	1.765	-14.34	19.60	0.0000 // E=	14.292
GLY A 876	0.139	1.722	2.871	0.574	-15.38	40.64	0.0000 // E=	30.564
GLU A 877	0.177	2.636	2.714	1.970	-23.50	-10.69	0.0000 // E=	-26.693
VAL A 878	0.305	2.146	5.306	0.580	-23.93	-1.31	0.0000 // E=	-16.902
VAL A 879	0.880	2.199	2.526	2.723	-36.93	-17.09	0.0000 // E=	-45.691
ALA A 880	0.097	0.277	4.534	0.312	-32.83	-13.36	0.0000 // E=	-40.970
VAL A 881	0.846	1.526	2.149	1.176	-35.41	-17.62	0.0000 // E=	-47.328
LYSH A 882	0.364	3.032	5.684	0.687	-45.13	-21.01	0.0000 // E=	-56.375
LYSH A 883	0.455	4.881	16.395	0.929	-39.86	-19.62	0.0000 // E=	-36.819
LEU A 884	0.347	3.378	4.546	3.580	-36.34	1.22	0.0000 // E=	-23.262
GLN A 885	1.284	8.035	15.950	1.856	-28.62	-164.69	0.0000 // E=	-166.177
HISA A 886	0.871	4.088	2.215	0.497	-15.60	0.74	0.0000 // E=	-7.187

CamScanner

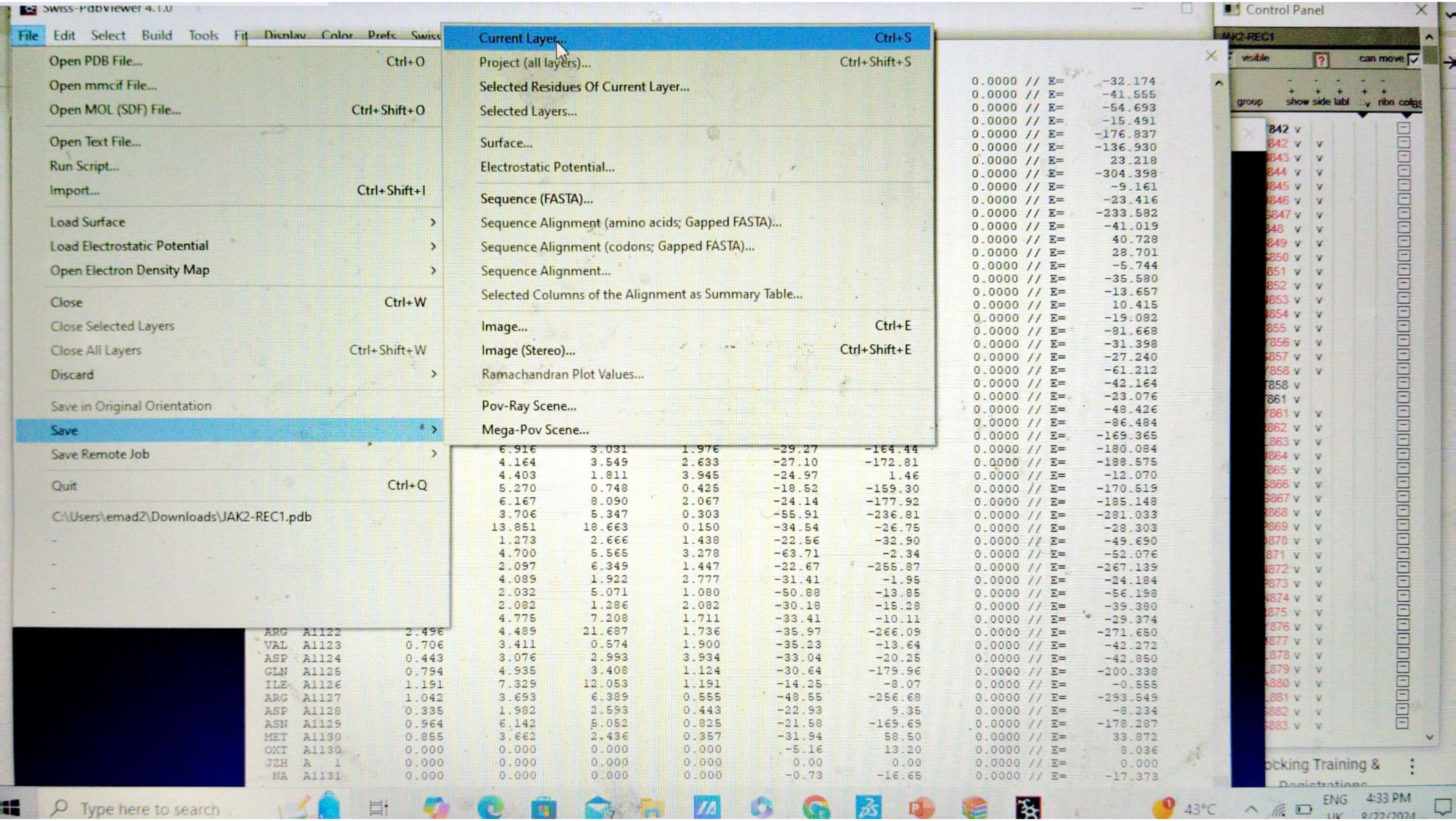
Swiss-PdbViewer 4.1.0
File Edit Select Build Tools Fit Display Color Prefs Swiss
Open PDB File... Ctrl+O
Open mmcif File...
Open MOL (SDF) File... Ctrl+Shift+O
Open Text File...
Run Script...
Import... Ctrl+Shift+I
Load Surface
Load Electrostatic Potential
Open Electron Density Map
Close Ctrl+W
Close Selected Layers
Close All Layers Ctrl+Shift+W
Discard
Save in Original Orientation
Save
Save Remote Job
Quit Ctrl+Q
C:\Users\emad2\Downloads\JAK2-REC1.pdb
Current Layer... Ctrl+S
Project (all layers)... Ctrl+Shift+S
Selected Residues Of Current Layer...
Selected Layers...
Surface...
Electrostatic Potential...
Sequence (FASTA)...
Sequence Alignment (amino acids; Gapped FASTA)...
Sequence Alignment (codons; Gapped FASTA)...
Sequence Alignment...
Selected Columns of the Alignment as Summary Table...
Image... Ctrl+E
Image (Stereo)... Ctrl+Shift+E
Ramachandran Plot Values...
Pov-Ray Scene...
Mega-Pov Scene...
Control Panel
JAK2-REC1
visible
can move
group
show side labl
ribn col
Type here to search
43°C
ENG
4:33 PM

CamScanner

JAK2-REC2.pdb - WordPad

```
64
SPDBVg    64    64    64    64    64    64    64    64    64
64
SPDBVg    64    64    64    64    64    64    64    64    64
64
SPDBVg    64    64    64    64    64    64    64    64    64
64
SPDBVg    64    64    64    64    64    64    64    64    64
64
SPDBVg    64    64    64    64    64    64    64    64    64
64
SPDBVg    64    64    64    64    64    64    64    64    64
64
SPDBVg    64    64    64    64    64    64    64    64    64
64
SPDBVg    64    64    64    64    64    64    64    64    64
64
SPDBVg    64    64    64    64    64    64    64    64    64
64
SPDBVg    64    64    64    64    64    64    64    64    64
64
SPDBVg    64    64    64    64    64    64    64    64    64
64
SPDBVg    64    64    64    64    64    64    64    64    64
64
SPDBVg    64    64    64    64    64    64    64    64    64
SPDBVi          1 1 1 0 1 0 1 1 0 1 0 1 1 0  0
SPDBVp          0
END
```

2.6. Molecular dynamics simulation

More details about MD simulation, software used, gromacs codes, and the estimated parameters are shown below

Problems occurred during gromacs processing were solved using https://manual.gromacs.org/documentation/current/user-guide/run-time-errors.html

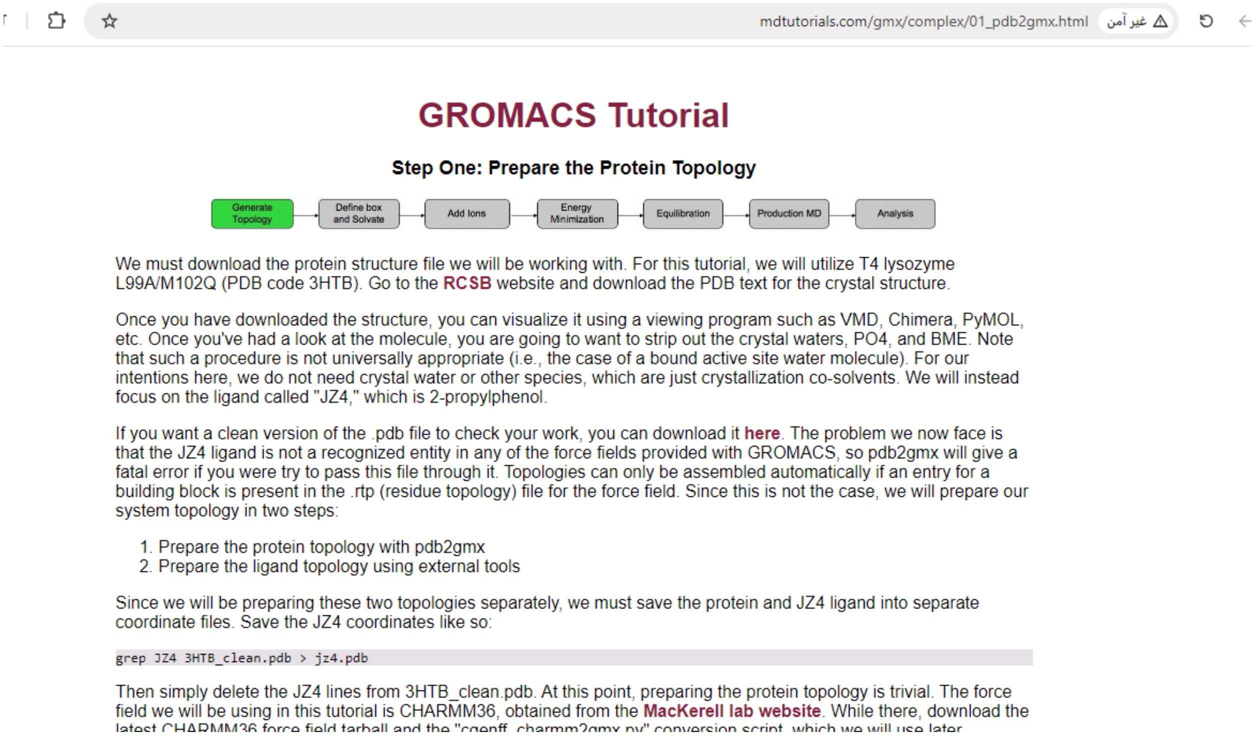

GROMACS Tutorial

Step One: Prepare the Protein Topology

We must download the protein structure file we will be working with. For this tutorial, we will utilize T4 lysozyme L99A/M102Q (PDB code 3HTB). Go to the **RCSB** website and download the PDB text for the crystal structure.

Once you have downloaded the structure, you can visualize it using a viewing program such as VMD, Chimera, PyMOL, etc. Once you've had a look at the molecule, you are going to want to strip out the crystal waters, PO4, and BME. Note that such a procedure is not universally appropriate (i.e., the case of a bound active site water molecule). For our intentions here, we do not need crystal water or other species, which are just crystallization co-solvents. We will instead focus on the ligand called "JZ4," which is 2-propylphenol.

If you want a clean version of the .pdb file to check your work, you can download it **here**. The problem we now face is that the JZ4 ligand is not a recognized entity in any of the force fields provided with GROMACS, so pdb2gmx will give a fatal error if you were try to pass this file through it. Topologies can only be assembled automatically if an entry for a building block is present in the .rtp (residue topology) file for the force field. Since this is not the case, we will prepare our system topology in two steps:

1. Prepare the protein topology with pdb2gmx
2. Prepare the ligand topology using external tools

Since we will be preparing these two topologies separately, we must save the protein and JZ4 ligand into separate coordinate files. Save the JZ4 coordinates like so:

```
grep JZ4 3HTB_clean.pdb > jz4.pdb
```

Then simply delete the JZ4 lines from 3HTB_clean.pdb. At this point, preparing the protein topology is trivial. The force field we will be using in this tutorial is CHARMM36, obtained from the **MacKerell lab website**. While there, download the latest CHARMM36 force field tarball and the "cgenff_charmm2gmx.py" conversion script, which we will use later

INSTALL UBUNTU for windows

Commands:

sudo apt update

sudo apt upgrade

sudo apt install gcc

sudo apt install cmake

sudo apt install build-essential

sudo apt install libfftw3-dev OR

sudo apt-get install -y libfftw3-dev

ONCE THE ABOVE COMMANDS ARE INTERED AND LINUX SYSTEM IS UPDATED, PROCEED TO INSTALL GROMACS

Install google chrome

get https://dl.google.com/linux/direct/google-chrome-stable_current_amd64.deb

sudo apt install ./google-chrome-stable_current_amd64.deb

Install Chimera

Download the set-up file from "https://www.cgl.ucsf.edu/chimera/download.html"

Move the file to desired folder.

In tht folder open terminal

Command:

ls (to check the name of setup file)

chmod +x CHIMERA-INSTALLER.bin

./CHIMERA-INSTALLER.bin

Install-GRACE

sudo apt-get install grace

MANNUAL-GROMACS-COMPILATION

Download Gromacs:

ftp://ftp.gromacs.org/gromacs/gromacs-2023.tar.gz

Gromacs Compilation Process

tar xfz gromacs-2022.3.tar.gz

cd gromacs-2020.2

mkdir build

cd build

cmake .. -DGMX_GPU=CUDA -DCUDA_TOOLKIT_ROOT_DIR=/usr/local/cuda

make

make check

sudo make install

source /usr/local/gromacs/bin/GMXRC

PREPARE LIGAND AND RECEPTOR IN CHIMERA

1. open the best pose ligand with the receptor Protein.pdb file

2. Delete the chain of protein, in the residual ligand, add hydrogens and save it as LIG.mol2 as 'LIG.mol2'

Open the LIG.mol2 file and in the second line

Correction to be made in LIG.mol2

Open LIG.mol2 by using gedit command or simply opening file in any text editor.

2.1. "@<TRIPOS>MOLECULE" make sure this is the first line in file

delete the header and empty space if you have to

2.2. "@<TRIPOS>MOLECULE” there will be name after this line maybe xxx.pdb

change it to LIG

2.3. bond orders "@<TRIPOS>BOND” arranged differently in each file arrange them in specific order to avoid errors use

perl sort_mol2_bonds.pl LIG.mol2 LIG.mol2 script

3. Go to SwissParam "http://www.swissparam.ch/" and upload the 'Lig.mol2 file'

4. Download the .zip folder

5. Open the best pose ligand with the receptor .pdb file, delete ligand, Perform DockPrep of protein as save it as .pdb file as 'REC.pdb'

6. Make a working Folder for Gromacs, copy contents of the downloaded zip file into this folder, copy the DockPrep 'rec.pdb' in to working folder

7. Copy all the .mdp files into this working folder

8. Open the terminal in this working folder and proceed with Gromacs.

GROMACS UBUNTU TUTORIAL

source /usr/local/gromacs/bin/GMXRC

gmx pdb2gmx -f REC.pdb -ignh

8 (CHARMM27)

1 (TIP3P)

gmx editconf -f LIG.pdb -o LIG.gro

gedit conf.gro LIG.gro

*(Copy content from 3rd line of lig.gro to the conf.gro file up to the 2nd last line)

*(Check the column number from where the lig.gro data ends (x) in conf.gro and replace the value in 2nd line by x-3)

*(Open file in chimera to check ligand and receptor)

EDIT THE FOLLOWING in topol.top

gedit topol.top

(add

; Include ligand topology

#include "LIG.itp"

below- Include forcefield parameters

#include "amberGS.ff/forcefield.itp")

AT THE BOTTOM OF THE SAME FILE PERFORM FOLLOWING CHANGES

(add LIG 1

align exactly below-

Protein_chain_E   1)

-----EDIT THE FOLLOWING in lig.itp -----

gedit lig.itp

[moleculetype]

; Name nrexcl

lig_gmx2 3

TO

[moleculetype]

; Name nrexcl

LIG 3

gmx editconf -f conf.gro -d 1.0 -bt triclinic -o box.gro

gmx solvate -cp box.gro -cs spc216.gro -p topol.top -o box_sol.gro

gmx grompp -f ions.mdp -c box_sol.gro -p topol.top -o ION.tpr

gmx genion -s ION.tpr -p topol.top -conc 0.1 -neutral -o box_sol_ion.gro

15

gmx grompp -f EM.mdp -c box_sol_ion.gro -p topol.top -o EM.tpr

gmx mdrun -v -deffnm EM

gedit nvt.mdp

Make index files

gmx make_ndx -f LIG.gro -o index_LIG.ndx

> 0 & ! a H*

> q

gmx genrestr -f LIG.gro -n index_LIG.ndx -o posre_LIG.itp -fc 1000 1000 1000

> select group "3"

Open topol.top file

at the end of the document

after

"; Include Position restraint file

#ifdef POSRES

#include "posre.itp"

#endif

"Here"

add this

; Ligand position restraints

#ifdef POSRES

#include "posre_LIG.itp"

#endif

Again, Make other Index file for System

gmx make_ndx -f EM.gro -o index.ndx

> 1 | 13

> q

[NVT]

gedit NVT.mdp (This file is already modified)

gmx grompp -f NVT.mdp -c EM.gro -r EM.gro -p topol.top -n index.ndx -maxwarn 2 -o NVT.tpr

gmx mdrun -deffnm NVT

[NPT]

gedit NPT.mdp (This file is already modified)

gmx grompp -f NPT.mdp -c NVT.gro -r NVT.gro -p topol.top -n index.ndx -maxwarn 2 -o NPT.tpr

gmx mdrun -deffnm NPT

[FINAL MD RUN/PRODUCTION]

gedit NPT.mdp (Change MD RUN TIME as per your need)

gmx grompp -f MD.mdp -c NPT.gro -t NPT.cpt -p topol.top -n index.ndx -maxwarn 2 -o MD.tpr

gmx mdrun -deffnm MD

[Recentering and Rewrapping Coordinates]

gmx trjconv -s MD.tpr -f MD.xtc -o MD_center.xtc -center -pbc mol -ur compact

#Choose "Protein" for centering and "System" for output.

#To extract the first frame (t = 0 ns) of the trajectory, use trjconv -dump with the recentered trajectory:

gmx trjconv -s MD.tpr -f MD_center.xtc -o start.pdb -dump 0

RMSD Calculations

gmx rms -s MD.tpr -f MD_center.xtc -o rmsd.xvg

gmx rms -s MD.tpr -f MD_center.xtc -o rmsd.xvg -tu ns

4

13

#(Select appropriate 2 options one by one and then open the output files in Grace) Select Backbone and then LIG

xmgrace rmsd.xvg

------RMSF Calculations-----

gmx rmsf -s MD.tpr -f MD_center.xtc -o rmsf.xvg

4

(Select appropriate Backbone open the output files in Grace)

xmgrace output.xvg

h-bonds

gmx hbond -s MD.tpr -f MD_center.xtc -num hb.xvg

gmx hbond -s MD.tpr -f MD_center.xtc -num hb.xvg -tu ns

1

13

xmgrace hb.xvg

Gyration Radius

gmx gyrate -s MD.tpr -f MD_center.xtc -o gyrate1.xvg

#Choose the group of your choice

xmgrace gyrate1.xvg

ENERGY Calculations

gmx energy -f MD.edr -o energy1.xvg

#Choose the option of your choice

xmgrace -nxy energy1.xvg

Representative image from the Ubuntu software, slides from the software installation and final production of parameters shown at results section

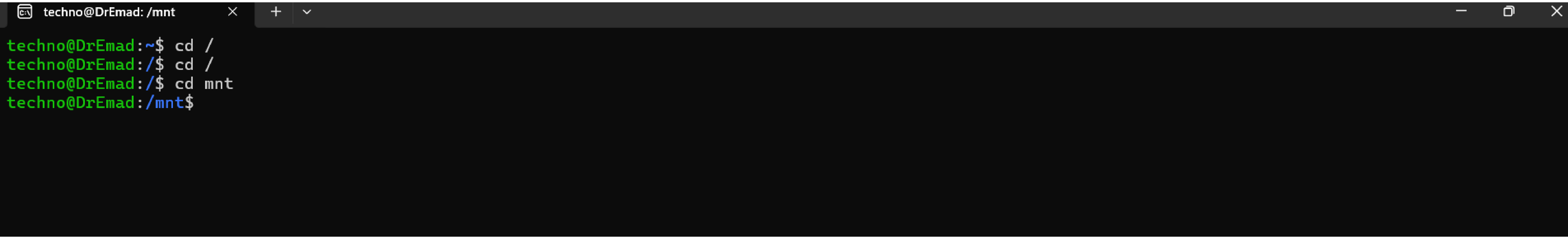

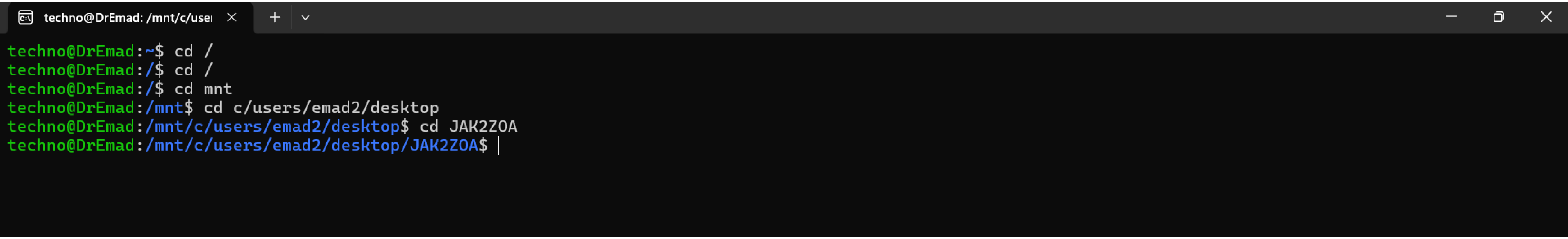

techno@DrEmad: /mnt/c/user

```
GROMACS is free software; you can redistribute it and/or modify it
under the terms of the GNU Lesser General Public License
as published by the Free Software Foundation; either version 2.1
of the License, or (at your option) any later version.

GROMACS:      gmx rms, version 2021.4-Ubuntu-2021.4-2
Executable:   /usr/bin/gmx
Data prefix:  /usr
Working dir:  /mnt/c/users/emad2/desktop/JAK2ZOA
Command line:
  gmx rms -s MD.tpr -f MD_center.xtc -o rmsd.xvg -tu ns

Reading file MD.tpr, VERSION 2021.4-Ubuntu-2021.4-2 (single precision)
Reading file MD.tpr, VERSION 2021.4-Ubuntu-2021.4-2 (single precision)
Select group for least squares fit
Group     0 (         System) has 36691 elements
Group     1 (        Protein) has  4661 elements
Group     2 (      Protein-H) has  2331 elements
Group     3 (        C-alpha) has   282 elements
Group     4 (       Backbone) has   846 elements
Group     5 (      MainChain) has  1127 elements
Group     6 (   MainChain+Cb) has  1393 elements
Group     7 (    MainChain+H) has  1399 elements
Group     8 (      SideChain) has  3262 elements
Group     9 (    SideChain-H) has  1204 elements
Group    10 (    Prot-Masses) has  4661 elements
Group    11 (    non-Protein) has 32030 elements
Group    12 (          Other) has    37 elements
Group    13 (            LIG) has    37 elements
Group    14 (             NA) has    23 elements
Group    15 (             CL) has    23 elements
Group    16 (          Water) has 31947 elements
Group    17 (            SOL) has 31947 elements
Group    18 (      non-Water) has  4744 elements
Group    19 (            Ion) has    46 elements
Group    20 (            LIG) has    37 elements
Group    21 (             NA) has    23 elements
Group    22 (             CL) has    23 elements
Group    23 ( Water_and_ions) has 31993 elements
Select a group:
```

techno@DrEmad: /mnt/c/user

```
Group    11 (    non-Protein) has 32030 elements
Group    12 (          Other) has    37 elements
Group    13 (            LIG) has    37 elements
Group    14 (             NA) has    23 elements
Group    15 (             CL) has    23 elements
Group    16 (          Water) has 31947 elements
Group    17 (            SOL) has 31947 elements
Group    18 (      non-Water) has  4744 elements
Group    19 (            Ion) has    46 elements
Group    20 (            LIG) has    37 elements
Group    21 (             NA) has    23 elements
Group    22 (             CL) has    23 elements
Group    23 ( Water_and_ions) has 31993 elements
Select a group: 4
Selected 4: 'Backbone'
Select group for RMSD calculation
Group     0 (         System) has 36691 elements
Group     1 (        Protein) has  4661 elements
Group     2 (      Protein-H) has  2331 elements
Group     3 (        C-alpha) has   282 elements
Group     4 (       Backbone) has   846 elements
Group     5 (      MainChain) has  1127 elements
Group     6 (   MainChain+Cb) has  1393 elements
Group     7 (    MainChain+H) has  1399 elements
Group     8 (      SideChain) has  3262 elements
Group     9 (    SideChain-H) has  1204 elements
Group    10 (    Prot-Masses) has  4661 elements
Group    11 (    non-Protein) has 32030 elements
Group    12 (          Other) has    37 elements
Group    13 (            LIG) has    37 elements
Group    14 (             NA) has    23 elements
Group    15 (             CL) has    23 elements
Group    16 (          Water) has 31947 elements
Group    17 (            SOL) has 31947 elements
Group    18 (      non-Water) has  4744 elements
Group    19 (            Ion) has    46 elements
Group    20 (            LIG) has    37 elements
Group    21 (             NA) has    23 elements
[illegible]
```

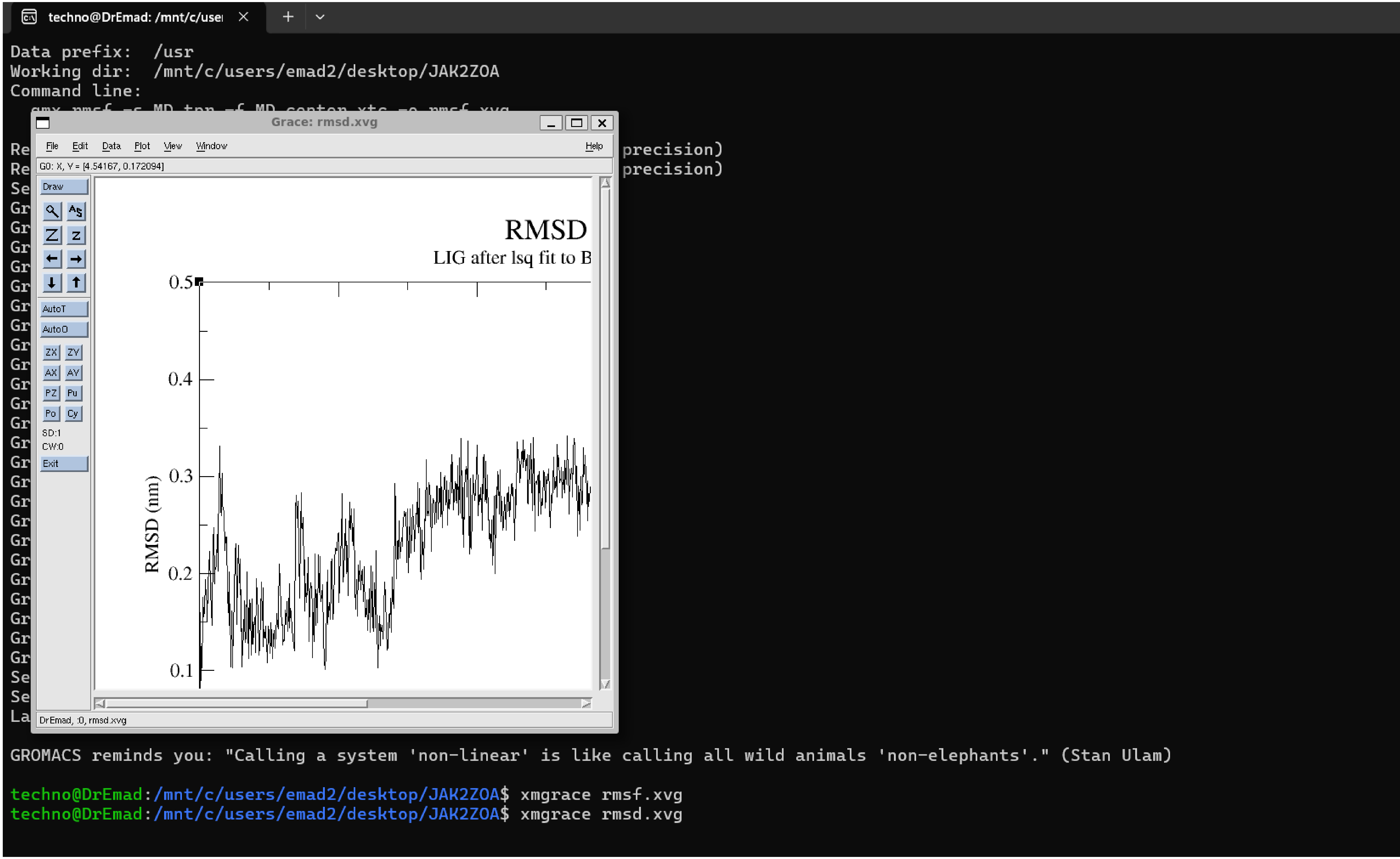
techno@DrEmad: /mnt/c/user
Data prefix: /usr
Working dir: /mnt/c/users/emad2/desktop/JAK2ZOA
Command line:
precision)
precision)
Grace: rmsd.xvg
File Edit Data Plot View Window Help
G0: X, Y = [4.54167, 0.172094]
Draw
AutoT
AutoO
ZX ZY
AX AY
PZ Pu
Po Cy
SD:1
CW:0
Exit
RMSD
LIG after lsq fit to B
RMSD (nm)
0.5
0.4
0.3
0.2
0.1
DrEmad, :0, rmsd.xvg
GROMACS reminds you: "Calling a system 'non-linear' is like calling all wild animals 'non-elephants'." (Stan Ulam)
techno@DrEmad:/mnt/c/users/emad2/desktop/JAK2ZOA$ xmgrace rmsf.xvg
techno@DrEmad:/mnt/c/users/emad2/desktop/JAK2ZOA$ xmgrace rmsd.xvg

```
techno@DrEmad: /mnt/c/use

GROMACS is free software; you can redistribute it and/or modify it
under the terms of the GNU Lesser General Public License
as published by the Free Software Foundation; either version 2.1
of the License, or (at your option) any later version.

GROMACS:      gmx rmsf, version 2021.4-Ubuntu-2021.4-2
Executable:   /usr/bin/gmx
Data prefix:  /usr
Working dir:  /mnt/c/users/emad2/desktop/JAK2ZOA
Command line:
  gmx rmsf -s MD.tpr -f MD_center.xtc -o rmsf.xvg

Reading file MD.tpr, VERSION 2021.4-Ubuntu-2021.4-2 (single precision)
Reading file MD.tpr, VERSION 2021.4-Ubuntu-2021.4-2 (single precision)
Select group(s) for root mean square calculation
Group     0 (         System) has 36691 elements
Group     1 (        Protein) has  4661 elements
Group     2 (      Protein-H) has  2331 elements
Group     3 (        C-alpha) has   282 elements
Group     4 (       Backbone) has   846 elements
Group     5 (      MainChain) has  1127 elements
Group     6 (   MainChain+Cb) has  1393 elements
Group     7 (    MainChain+H) has  1399 elements
Group     8 (      SideChain) has  3262 elements
Group     9 (    SideChain-H) has  1204 elements
Group    10 (    Prot-Masses) has  4661 elements
Group    11 (    non-Protein) has 32030 elements
Group    12 (          Other) has    37 elements
Group    13 (            LIG) has    37 elements
Group    14 (             NA) has    23 elements
Group    15 (             CL) has    23 elements
Group    16 (          Water) has 31947 elements
Group    17 (            SOL) has 31947 elements
Group    18 (      non-Water) has  4744 elements
Group    19 (            Ion) has    46 elements
Group    20 (            LIG) has    37 elements
Group    21 (             NA) has    23 elements
Group    22 (             CL) has    23 elements
Group    23 ( Water_and_ions) has 31993 elements
Select a group: 4
```

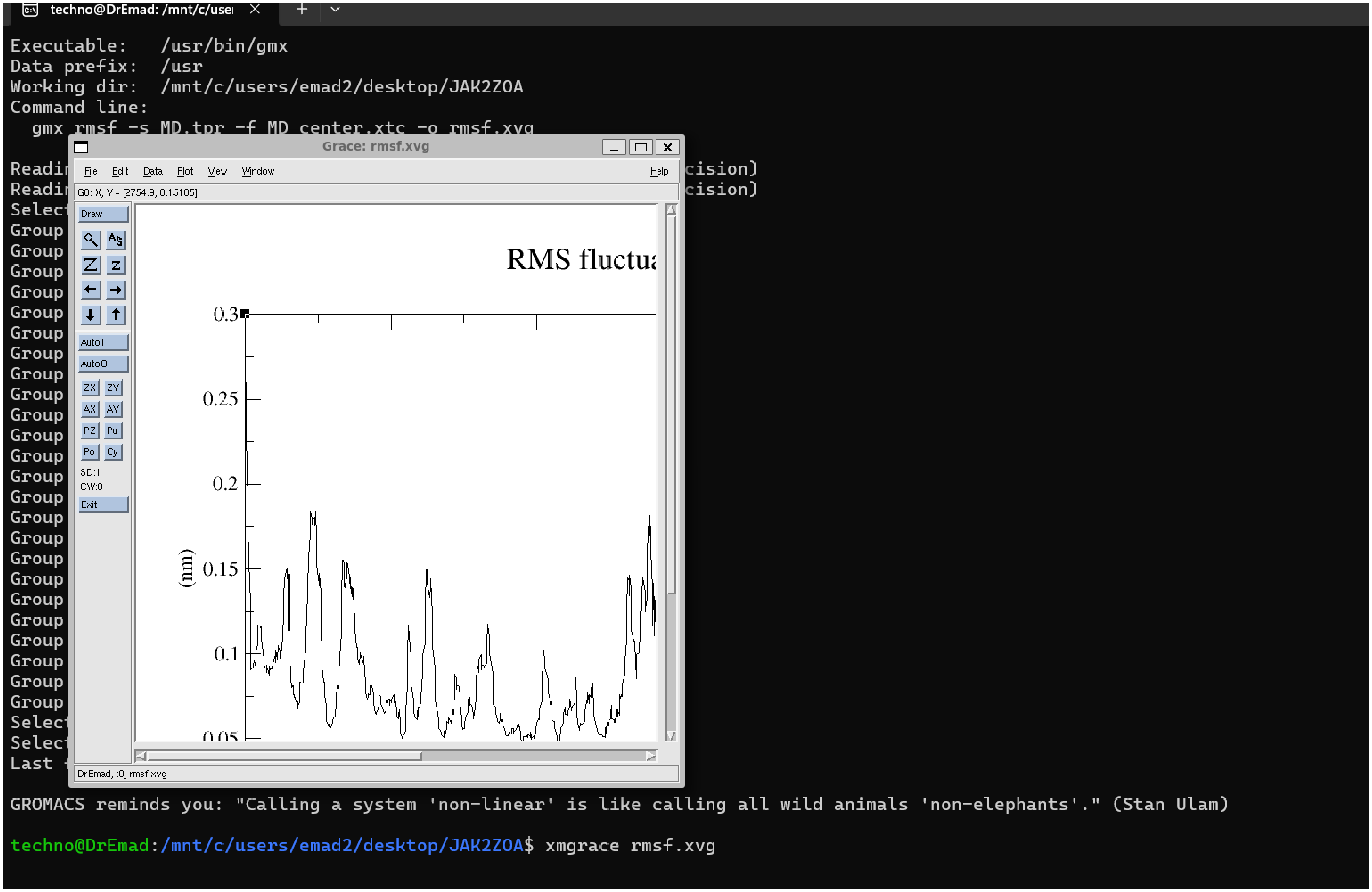

techno@DrEmad: /mnt/c/user
Executable: /usr/bin/gmx
Data prefix: /usr
Working dir: /mnt/c/users/emad2/desktop/JAK2ZOA
Command line:
gmx rmsf -s MD.tpr -f MD_center.xtc -o rmsf.xvg
Grace: rmsf.xvg
File Edit Data Plot View Window Help
G0: X, Y = [2754.9, 0.15105]
Draw
AutoT
AutoO
ZX ZY
AX AY
PZ Pu
Po Cy
SD:1
CW:0
Exit
RMS fluctu
0.3
0.25
0.2
0.15
0.1
0.05
(nm)
DrEmad, :0, rmsf.xvg
cision)
cision)
GROMACS reminds you: "Calling a system 'non-linear' is like calling all wild animals 'non-elephants'." (Stan Ulam)
techno@DrEmad:/mnt/c/users/emad2/desktop/JAK2ZOA$ xmgrace rmsf.xvg

```
techno@DrEmad: /mnt/c/use

GROMACS is free software; you can redistribute it and/or modify it
under the terms of the GNU Lesser General Public License
as published by the Free Software Foundation; either version 2.1
of the License, or (at your option) any later version.

GROMACS:      gmx hbond, version 2021.4-Ubuntu-2021.4-2
Executable:   /usr/bin/gmx
Data prefix:  /usr
Working dir:  /mnt/c/users/emad2/desktop/JAK2ZOA
Command line:
  gmx hbond -s MD.tpr -f MD_center.xtc -num hb.xvg

Reading file MD.tpr, VERSION 2021.4-Ubuntu-2021.4-2 (single precision)
Specify 2 groups to analyze:
Group     0 (         System) has 36691 elements
Group     1 (        Protein) has  4661 elements
Group     2 (      Protein-H) has  2331 elements
Group     3 (        C-alpha) has   282 elements
Group     4 (       Backbone) has   846 elements
Group     5 (      MainChain) has  1127 elements
Group     6 (   MainChain+Cb) has  1393 elements
Group     7 (    MainChain+H) has  1399 elements
Group     8 (      SideChain) has  3262 elements
Group     9 (    SideChain-H) has  1204 elements
Group    10 (    Prot-Masses) has  4661 elements
Group    11 (    non-Protein) has 32030 elements
Group    12 (          Other) has    37 elements
Group    13 (            LIG) has    37 elements
Group    14 (             NA) has    23 elements
Group    15 (             CL) has    23 elements
Group    16 (          Water) has 31947 elements
Group    17 (            SOL) has 31947 elements
Group    18 (      non-Water) has  4744 elements
Group    19 (            Ion) has    46 elements
Group    20 (            LIG) has    37 elements
Group    21 (             NA) has    23 elements
Group    22 (             CL) has    23 elements
Group    23 ( Water_and_ions) has 31993 elements
Select a group: 1
Selected 1: 'Protein'
Select a group: 13
```

```
Select a group: 1
Selected 1: 'Protein'
Select a group: 13
Selected 13: 'LIG'
Checking for overlap in atoms between Protein and LIG
Calculating hydrogen bonds between Protein (4661 atoms) and LIG (37 atoms)
Found 425 donors and 843 acceptors
Reading frame       0 time    0.000
Will do grid-search on 17x17x15 grid, rcut=0.34999999
Frame loop parallelized with OpenMP using 12 threads.
Last frame       1000 time 10000.000
Average number of hbonds per timeframe 1.098 out of 179138 possible

GROMACS reminds you: "I invented the term 'Object-Oriented', and I can tell you I did not have C++ in mind." (Alan Kay, author of Smalltalk)
```

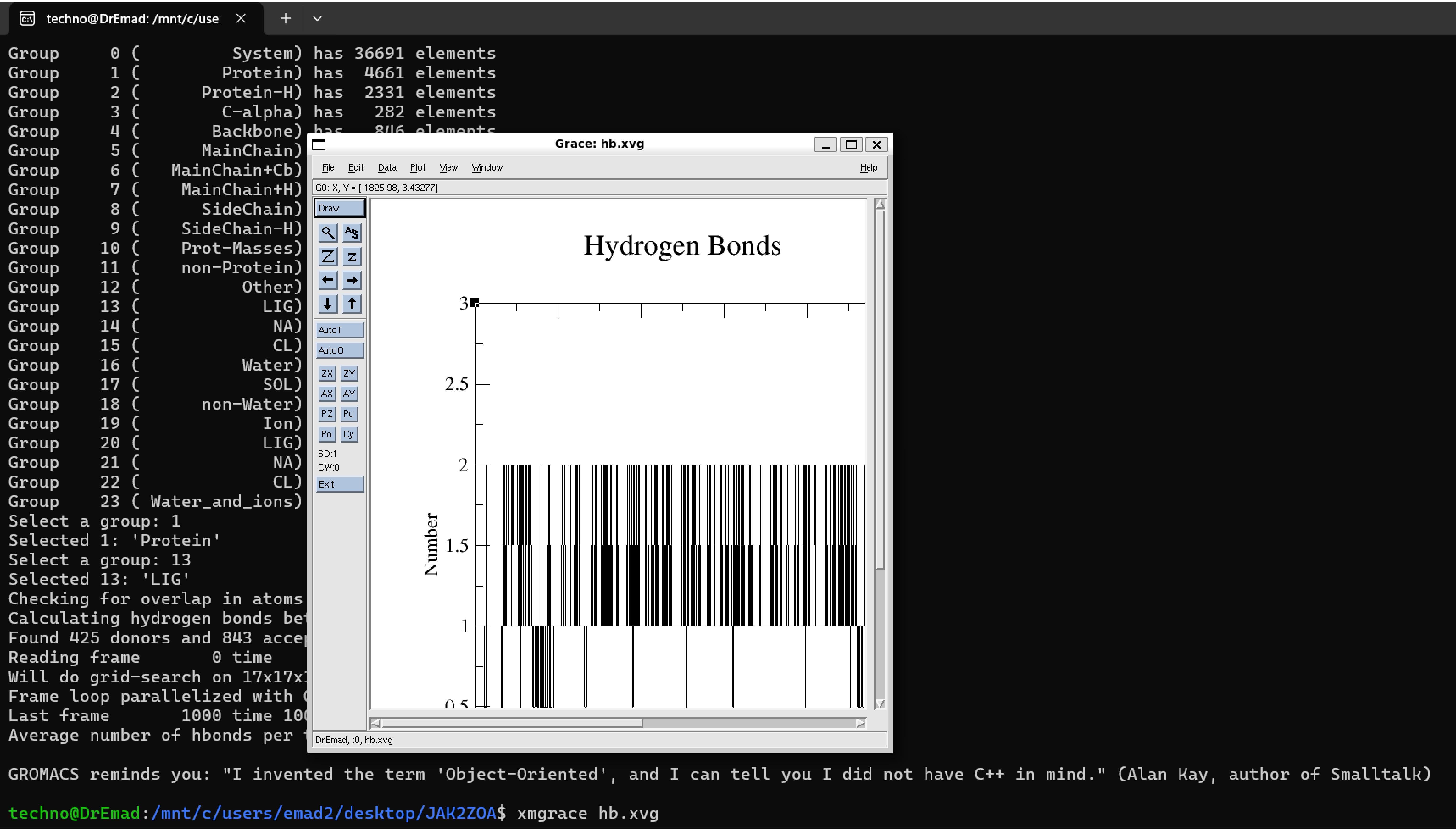
techno@DrEmad: /mnt/c/user
Group 0 (System) has 36691 elements
Group 1 (Protein) has 4661 elements
Group 2 (Protein-H) has 2331 elements
Group 3 (C-alpha) has 282 elements
Group 4 (Backbone)
Group 5 (MainChain)
Group 6 (MainChain+Cb)
Group 7 (MainChain+H)
Group 8 (SideChain)
Group 9 (SideChain-H)
Group 10 (Prot-Masses)
Group 11 (non-Protein)
Group 12 (Other)
Group 13 (LIG)
Group 14 (NA)
Group 15 (CL)
Group 16 (Water)
Group 17 (SOL)
Group 18 (non-Water)
Group 19 (Ion)
Group 20 (LIG)
Group 21 (NA)
Group 22 (CL)
Group 23 (Water_and_ions)
Select a group: 1
Selected 1: 'Protein'
Select a group: 13
Selected 13: 'LIG'
Checking for overlap in atoms
Calculating hydrogen bonds be
Found 425 donors and 843 acce
Reading frame 0 time
Will do grid-search on 17x17x
Frame loop parallelized with
Last frame 1000 time 10
Average number of hbonds per
GROMACS reminds you: "I invented the term 'Object-Oriented', and I can tell you I did not have C++ in mind." (Alan Kay, author of Smalltalk)
techno@DrEmad:/mnt/c/users/emad2/desktop/JAK2ZOA$ xmgrace hb.xvg
Grace: hb.xvg
File Edit Data Plot View Window Help
G0: X, Y = [-1825.98, 3.43277]
Draw
AutoT
AutoO
ZX ZY
AX AY
PZ Pu
Po Cy
SD:1
CW:0
Exit
Hydrogen Bonds
Number
3
2.5
2
1.5
1
DrEmad, :0, hb.xvg

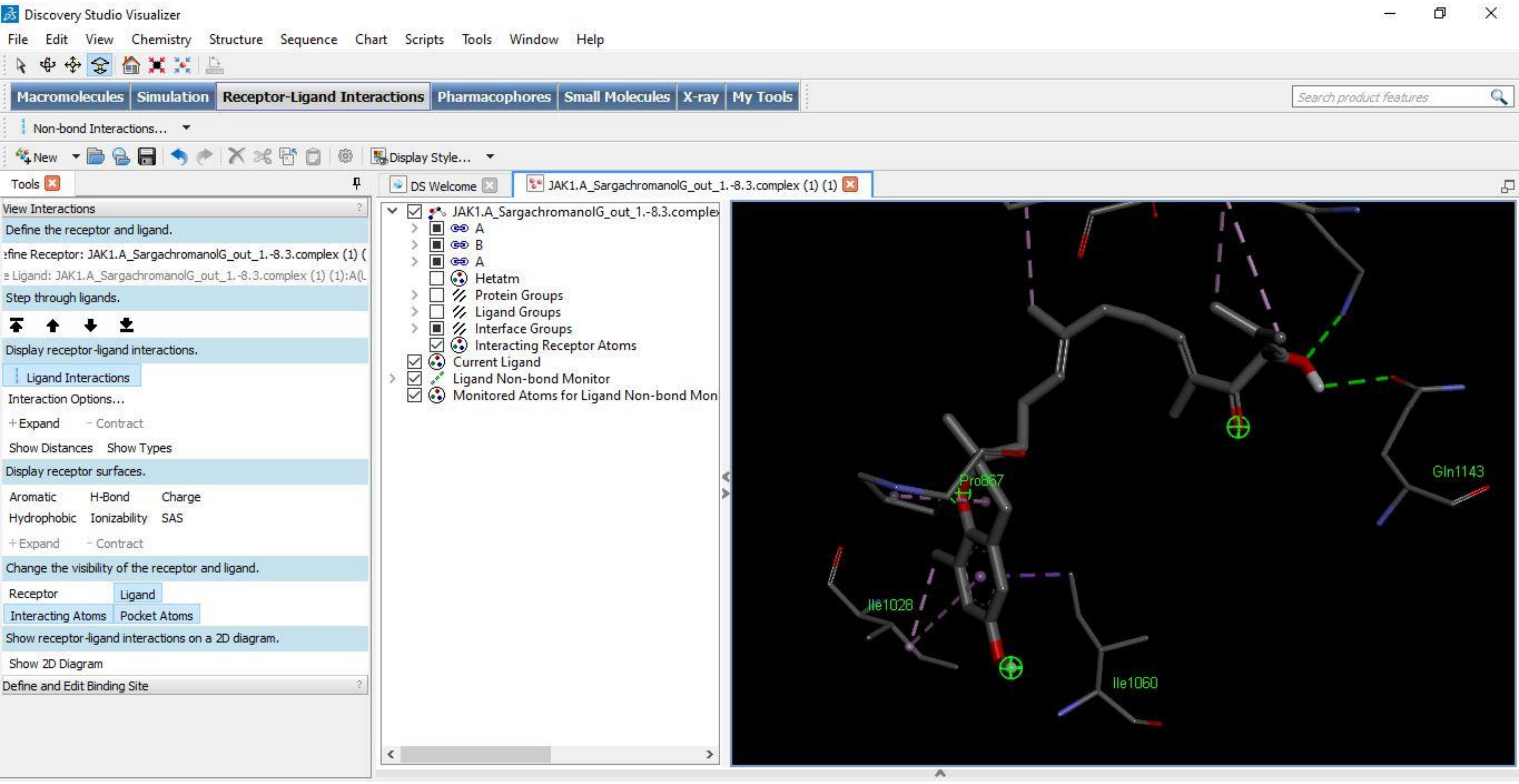
Discovery Studio Visualizer
File Edit View Chemistry Structure Sequence Chart Scripts Tools Window Help
Macromolecules Simulation Receptor-Ligand Interactions Pharmacophores Small Molecules X-ray My Tools
Search product features
Non-bond Interactions...
New
Display Style...
Tools
DS Welcome
JAK1.A_SargachromanolG_out_1.-8.3.complex (1) (1)
View Interactions
Define the receptor and ligand.
efine Receptor: JAK1.A_SargachromanolG_out_1.-8.3.complex (1) (
e Ligand: JAK1.A_SargachromanolG_out_1.-8.3.complex (1) (1):A(L
Step through ligands.
Display receptor-ligand interactions.
Ligand Interactions
Interaction Options...
+Expand
- Contract
Show Distances
Show Types
Display receptor surfaces.
Aromatic
H-Bond
Charge
Hydrophobic
Ionizability
SAS
+Expand
- Contract
Change the visibility of the receptor and ligand.
Receptor
Ligand
Interacting Atoms
Pocket Atoms
Show receptor-ligand interactions on a 2D diagram.
Show 2D Diagram
Define and Edit Binding Site
JAK1.A_SargachromanolG_out_1.-8.3.complex
A
B
A
Hetatm
Protein Groups
Ligand Groups
Interface Groups
Interacting Receptor Atoms
Current Ligand
Ligand Non-bond Monitor
Monitored Atoms for Ligand Non-bond Mon
Pro867
Gln1143
Ile1028
Ile1060

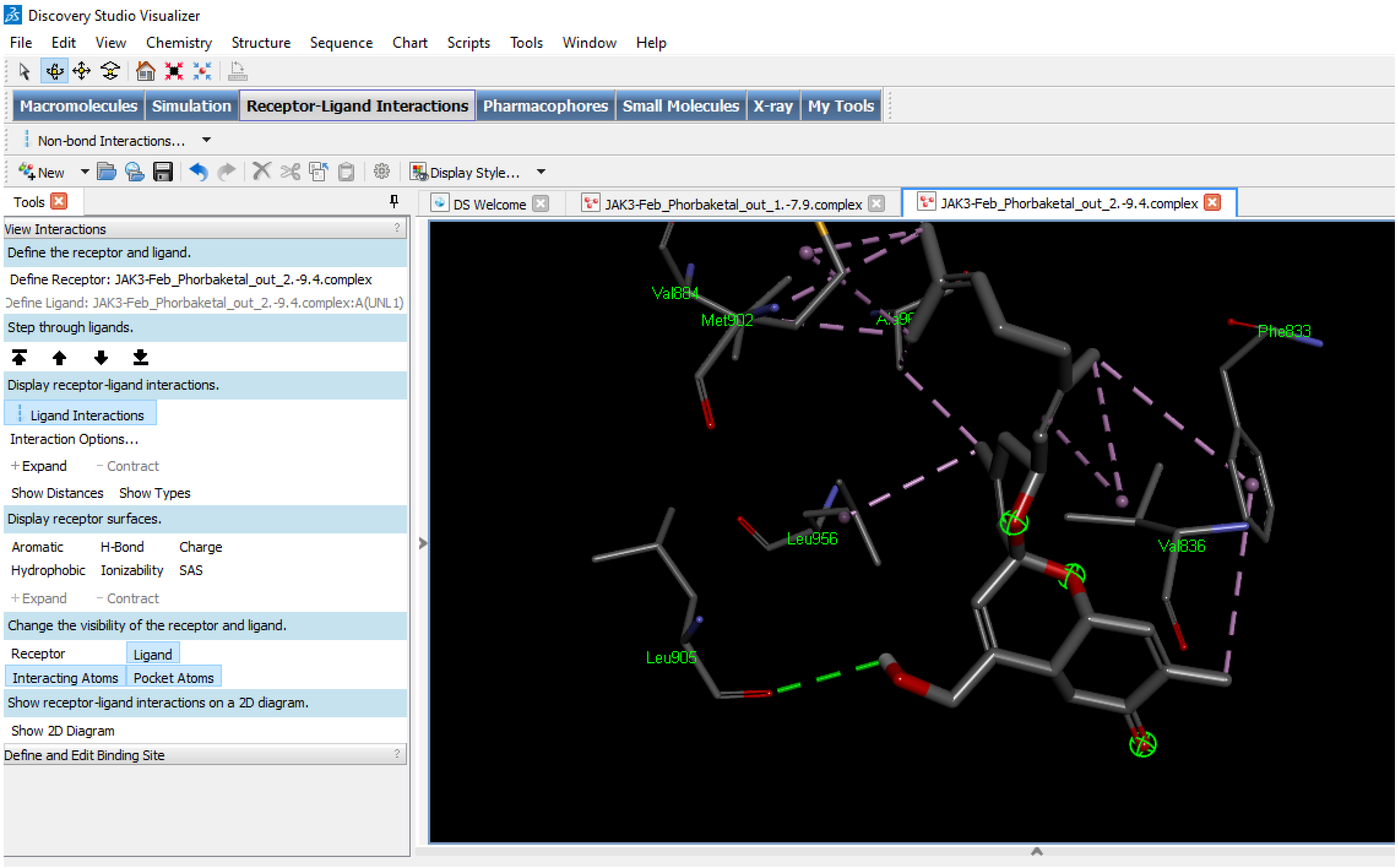

Discovery Studio Visualizer
File Edit View Chemistry Structure Sequence Chart Scripts Tools Window Help
Macromolecules
Simulation
Receptor-Ligand Interactions
Pharmacophores
Small Molecules
X-ray
My Tools
Non-bond Interactions...
New
Display Style...
Tools
DS Welcome
JAK3-Feb_Phorbaketal_out_1.-7.9.complex
JAK3-Feb_Phorbaketal_out_2.-9.4.complex
View Interactions
Define the receptor and ligand.
Define Receptor: JAK3-Feb_Phorbaketal_out_2.-9.4.complex
Define Ligand: JAK3-Feb_Phorbaketal_out_2.-9.4.complex:A(UNL1)
Step through ligands.
Display receptor-ligand interactions.
Ligand Interactions
Interaction Options...
+Expand
- Contract
Show Distances
Show Types
Display receptor surfaces.
Aromatic
H-Bond
Charge
Hydrophobic
Ionizability
SAS
+Expand
- Contract
Change the visibility of the receptor and ligand.
Receptor
Ligand
Interacting Atoms
Pocket Atoms
Show receptor-ligand interactions on a 2D diagram.
Show 2D Diagram
Define and Edit Binding Site
Val884
Met902
Phe833
Leu956
Val836
Leu905
