# Supplementary figures and images for: Marine Bioactive Molecules as Inhibitors of the Janus Kinases: A Comparative Molecular Docking and Molecular Dynamics Simulation Approach
Source: Curr Issues Mol Biol. 2024 Sep 23;46(9):10635–50. doi: 10.3390/cimb46090631 (PMC11430628; doi:10.3390/cimb46090631)

## Slide 1
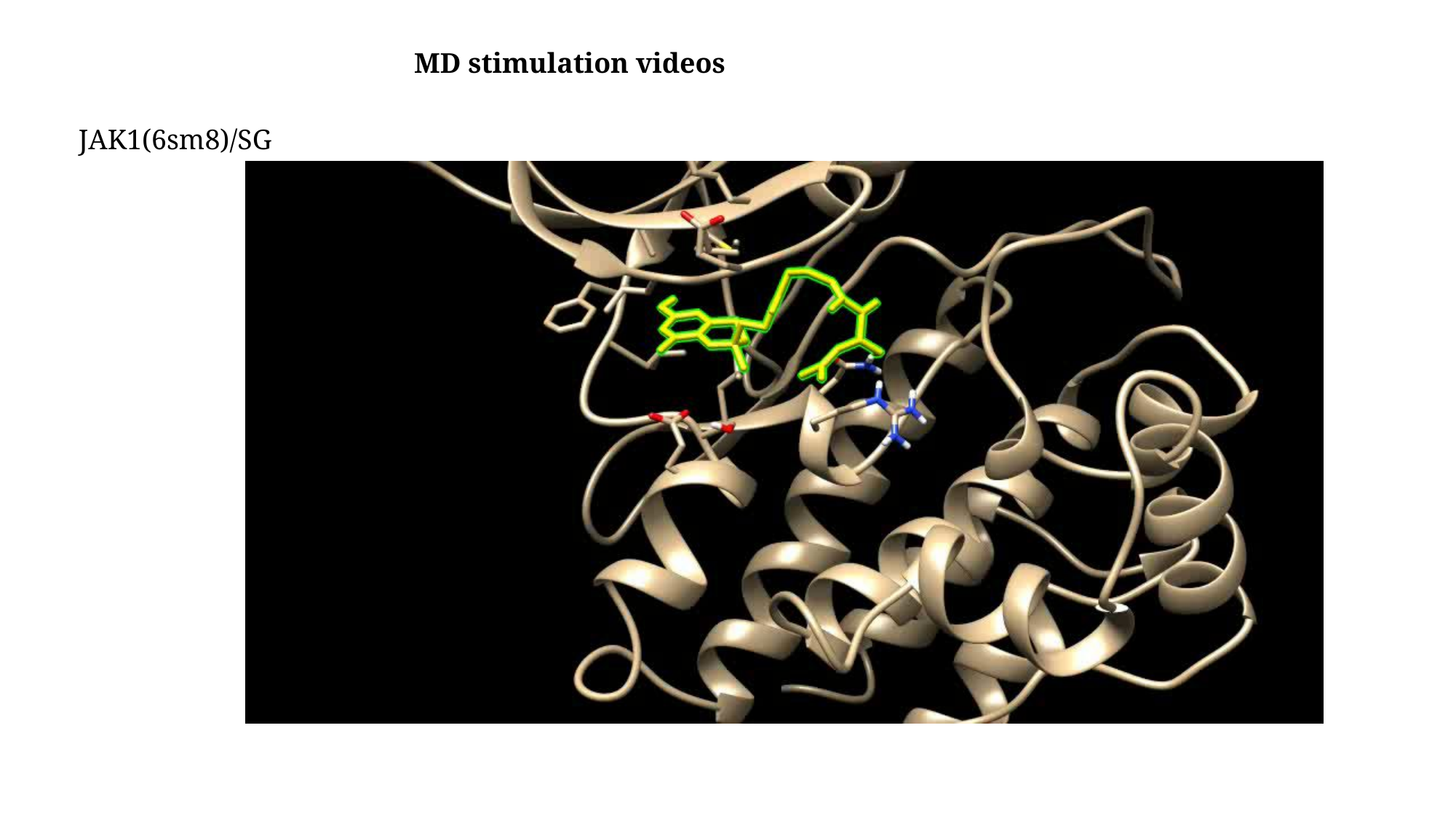

MD stimulation videos
JAK1(6sm8)/SG

## Slide 2
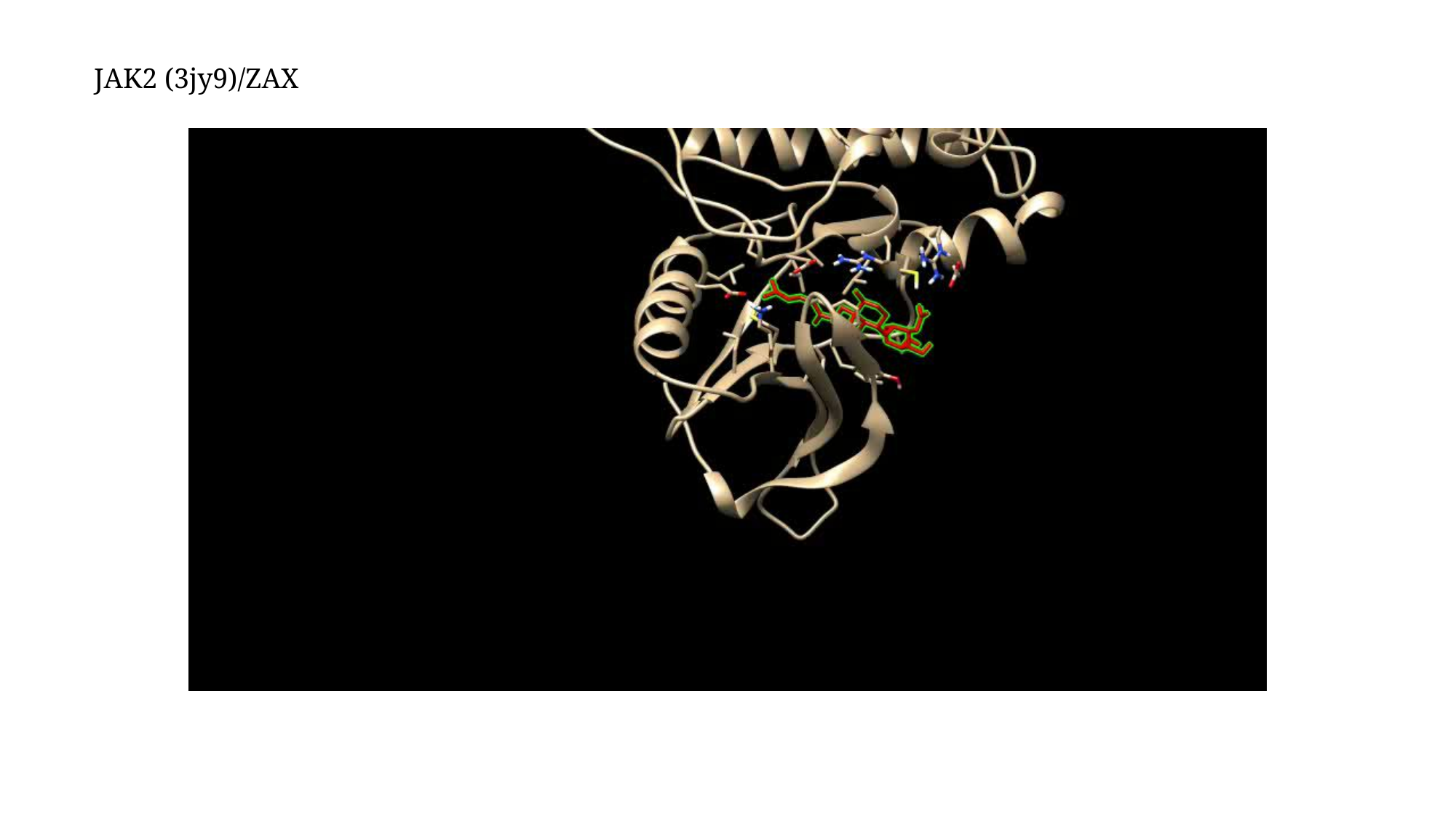

JAK2 (3jy9)/ZAX

## Slide 3
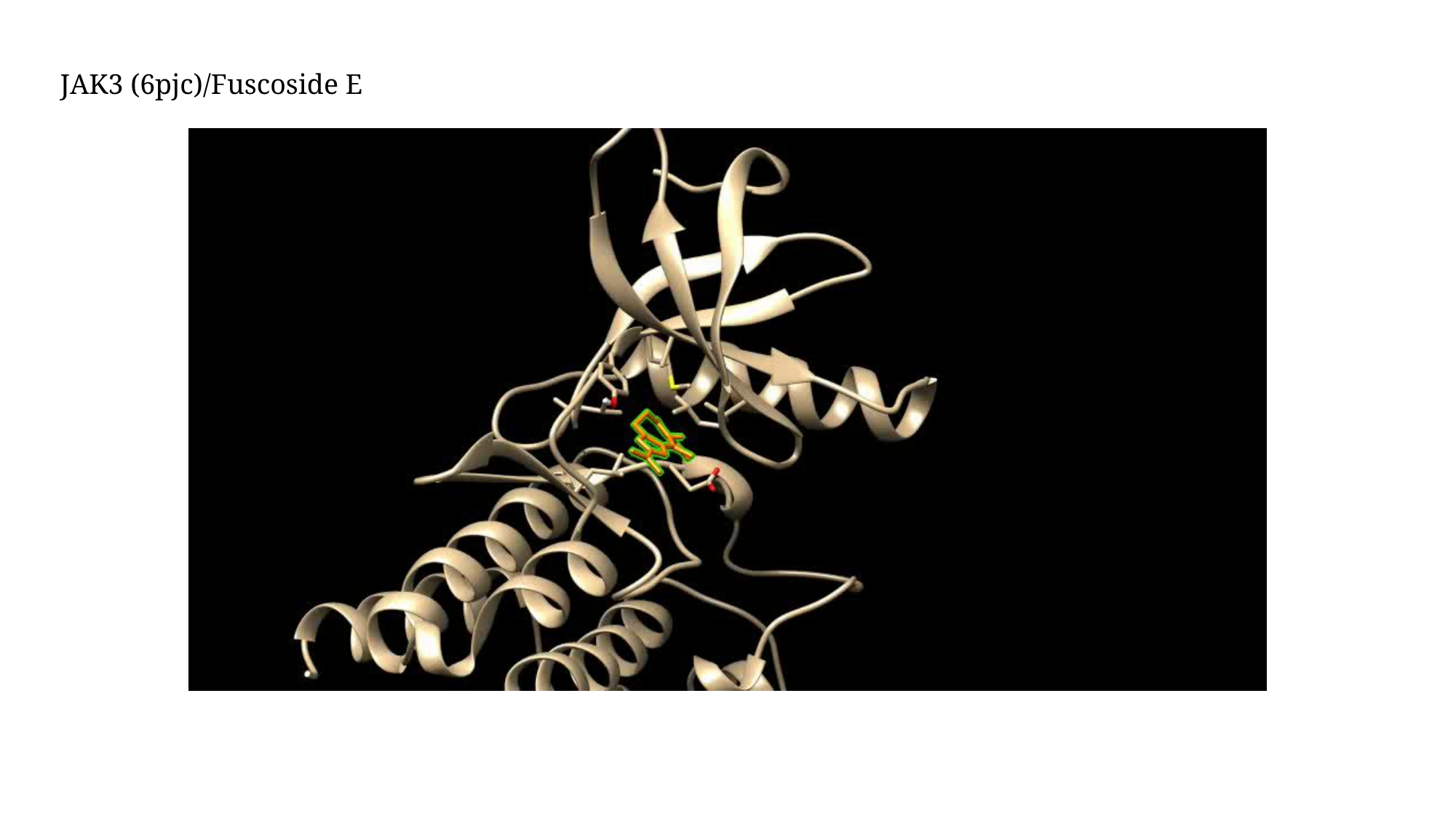

JAK3 (6pjc)/Fuscoside E

Supplement: Supplementary file 1 [file cimb-46-00631-s001.zip › cimb-3187994-supplementary.PPTX]
